# *Protein phosphatase 4 regulatory subunit 2 (PPP4R2)* is recurrently deleted in acute myeloid leukemia and required for efficient DNA double strand break repair

**DOI:** 10.18632/oncotarget.21119

**Published:** 2017-09-21

**Authors:** Julia K. Herzig, Lars Bullinger, Alpaslan Tasdogan, Philipp Zimmermann, Martin Schlegel, Veronica Teleanu, Daniela Weber, Frank G. Rücker, Peter Paschka, Anna Dolnik, Edith Schneider, Florian Kuchenbauer, Florian H. Heidel, Christian Buske, Hartmut Döhner, Konstanze Döhner, Verena I. Gaidzik

**Affiliations:** ^1^ Department of Internal Medicine III, University Hospital of Ulm, Ulm, Germany; ^2^ Institute of Immunology, Ulm University, Ulm, Germany; ^3^ Institute of Experimental Cancer Research, University Hospital of Ulm, Ulm, Germany; ^4^ Leibniz Institute on Aging–Fritz Lipmann Institute, Jena, Germany; ^5^ Innere Medizin II, Hämatologie und Onkologie, Universitätsklinikum Jena, Jena, Germany; ^6^ Current/Present address: Children's Medical Center Research Institute, UT Southwestern, Dallas, TX, USA

**Keywords:** AML, gene deletion, 3p, PPP4R2, DNA repair

## Abstract

We have previously identified a recurrent deletion at chromosomal band 3p14.1-p13 in patients with acute myeloid leukemia (AML). Among eight protein-coding genes, this microdeletion affects the *protein phosphatase 4 regulatory subunit 2* (*PPP4R2*), which plays an important role in DNA damage response (DDR). Investigation of mRNA expression during murine myelopoiesis determined that *Ppp4r2* is higher expressed in more primitive hematopoietic cells. *PPP4R2* expression in primary AML samples compared to healthy bone marrow was significantly lower, particularly in patients with 3p microdeletion or complex karyotype. To identify a functional role of PPP4R2 in hematopoiesis and leukemia, we genetically inactivated *Ppp4r2* by RNAi in murine hematopoietic stem and progenitor cells and murine myeloid leukemia. Furthermore, we ectopically expressed *PPP4R2* in a deficient human myeloid leukemic cell line. While *PPP4R2* is involved in DDR of both hematopoietic and leukemic cells, our findings indicate that *PPP4R2* deficiency impairs de-phosphorylation of phosphorylated key DDR proteins KRAB-domain associated protein 1 (pKAP1), histone variant H2AX (γH2AX), tumor protein P53 (pP53), and replication protein A2 (pRPA2). Potential impact of affected DNA repair processes in primary AML cases with regard to differential *PPP4R2* expression or 3p microdeletion is also supported by our results obtained by gene expression profiling and whole exome sequencing. Impaired DDR and increased DNA damage by *PPP4R2* suppression is one possible mechanism by which the 3p microdeletion may contribute to the pathogenesis of AML. Further studies are warranted to determine the potential benefit of inefficient DNA repair upon *PPP4R2* deletion to the development of therapeutic agents.

## INTRODUCTION

Recent developments in genomic techniques have tremendously improved our knowledge about the complex genomic architecture of acute myeloid leukemia (AML) and have shed further light on the genetic heterogeneity of the disease. Despite the increasing number of genetic abnormalities only a minority of these findings has entered clinical practice by displaying prognostic and predictive relevance used for risk-adapted therapeutic strategies [1–6]. Most genetic studies have been descriptive and do not clarify functional relevance of specific candidate genes located in recurrently altered genomic regions.

Using high-resolution single-nucleotide polymorphism (SNP) analyses we have previously identified a recurrent microdeletion at chromosomal band 3p14.1-p13 in cytogenetically normal (CN-) AML [7, 8]. In addition, we found this commonly deleted region (CDR) also in complex karyotype (CK-) AML [9]. The CDR spans a genomic region of 2 Mbp including eight protein-coding genes [*forkhead box P1* (*FOXP1*), *eukaryotic translation initiation factor 4E family member 3* (*EIF4E3*), *G protein-coupled receptor 27* (*GPR27*), *prokineticin 2* (*PROK2*), *RING1 and YY1 binding protein* (*RYBP*), *H/ACA ribonucleoprotein assembly factor* (*SHQ1*), *glucoside xylosyltransferase 2* (*GXYLT2*), and *protein phosphatase 4 regulatory subunit 2* (*PPP4R2*)] and one microRNA (miR-1284). Recurrent copy number loss with focus on the region of chromosome 3p was recently reported for patients with solid cancers like prostate and cervical cancer [10–14]. Several genes affected by the CDR, e.g. *FOXP1*, *RYBP*, and *SHQ1*, have been implicated to cooperate tumor suppressive functions that are related to apoptosis, proliferation, metastasis, and chemoradioresistance [10, 12, 14]. Furthermore, loss and downregulation of candidate genes within the CDR (e.g. *RYBP, SHQ1, FOXP1)* have been associated with poor clinical outcome in different types of solid tumors like cervical cancer, non-small cell lung cancer, hepatocellular carcinoma, or breast cancer [11, 12, 15, 16]. The human myeloid leukemic cell line MEG-01 carries a homozygous deletion of *PPP4R2* in addition to the heterozygous deletion of the other 3p candidate genes, which suggests a potential role of *PPP4R2* in hematopoiesis and leukemia development. Regulatory subunits like PPP4R2 govern the activity and substrate specificity of the catalytic subunit of the protein phosphatase 4 complex (PPP4) [17]. Previous studies highlighted a functional role of *PPP4R2* in cell development and differentiation, apoptosis, tumor progression, and DNA repair [18–27], which prompted us to focus the present work on the investigation of the candidate gene *PPP4R2*. Genetic inactivation studies identified that *PPP4R2* deficiency impedes efficient repair of DNA damage in human cells [23–25, 27] by regulating the de-phosphorylation of critical DNA damage response (DDR) proteins that are required for efficient DNA repair like the phosphorylated KRAB-domain associated protein 1 (pKAP1), the phosphorylated histone variant H2AX (γH2AX), or the phosphorylated replication protein A2 (pRPA2) [23–27]. Furthermore, depletion of *PPP4R2* resulted in elevated levels of DDR proteins γH2AX, pKAP1, or pRPA2, and has been associated with impaired homologous recombination- (HR) and non-homologous end-joining (NHEJ)-mediated DNA repair [23–27] representing the two major pathways that counteract DNA double strand breaks (DSB). DDR is an important mechanism to maintain genomic integrity, and is mediated by sequential phosphorylation of DDR proteins to recruit DNA repair and signaling proteins to the site of DNA damage [28]. The balance and termination upon activation of DDR signaling is carried out by protein phosphatases like PPP4.

The role of PPP4R2 in governing PPP4 and its de-phosphorylation activity has not been investigated in myelopoiesis and associated neoplasia so far. Here, we identified PPP4R2 as a critical regulator of cell survival and DDR signaling in normal hematopoietic and leukemic cells. Enhanced DNA damage as result of reduced *PPP4R2* expression might be one possible mechanism by which the 3p CDR contributes to the pathogenesis of AML.

## RESULTS

### Differential *PPP4R2* expression levels point to functional relevance during hematopoiesis and neoplasia

By screening the AML Study Group (AMLSG) metadatabase we were able to enlarge the cohort of AML patients encompassing the CDR at chromosomal band 3p14.1-p13 [7–9]. In total, we identified 10 AML patients harboring the 3p microdeletion within a normal karyotype, and additional 29 CK-AML cases with genomic loss at chromosome 3p affecting the CDR at 3p14.1-p13 including *PPP4R2* (Supplementary Table 1). Deletion of this region was validated by cytogenetic and/or fluorescence *in situ* hybridization (FISH) analysis as well as SNP array analyses [7, 9]. First, we assessed whether *PPP4R2* is mutated in AML patients (Supplementary Table 2). We screened a cohort of primary AML patient samples (*n* = 89) including CN-AML patients with 3p CDR for mutations in *PPP4R2*. This cohort was selected based on gene expression profiling (GEP) data and included patients with high (*n* = 30), intermediate (*n* = 29), and low (*n* = 30) global *PPP4R2* expression. Despite a polymorphism in exon 8 (HetC+Tpos1, rs61188513) in 12% of patients (*n* = 11; high expression, *n* = 5; intermediate expression, *n* = 1; low expression, *n* = 5) and several polymorphisms at intron/exon boundaries, no *PPP4R2* mutations were detected in this patient cohort. To our knowledge, none of the polymorphisms have been linked to leukemia or cancer so far.

**Figure 1:**
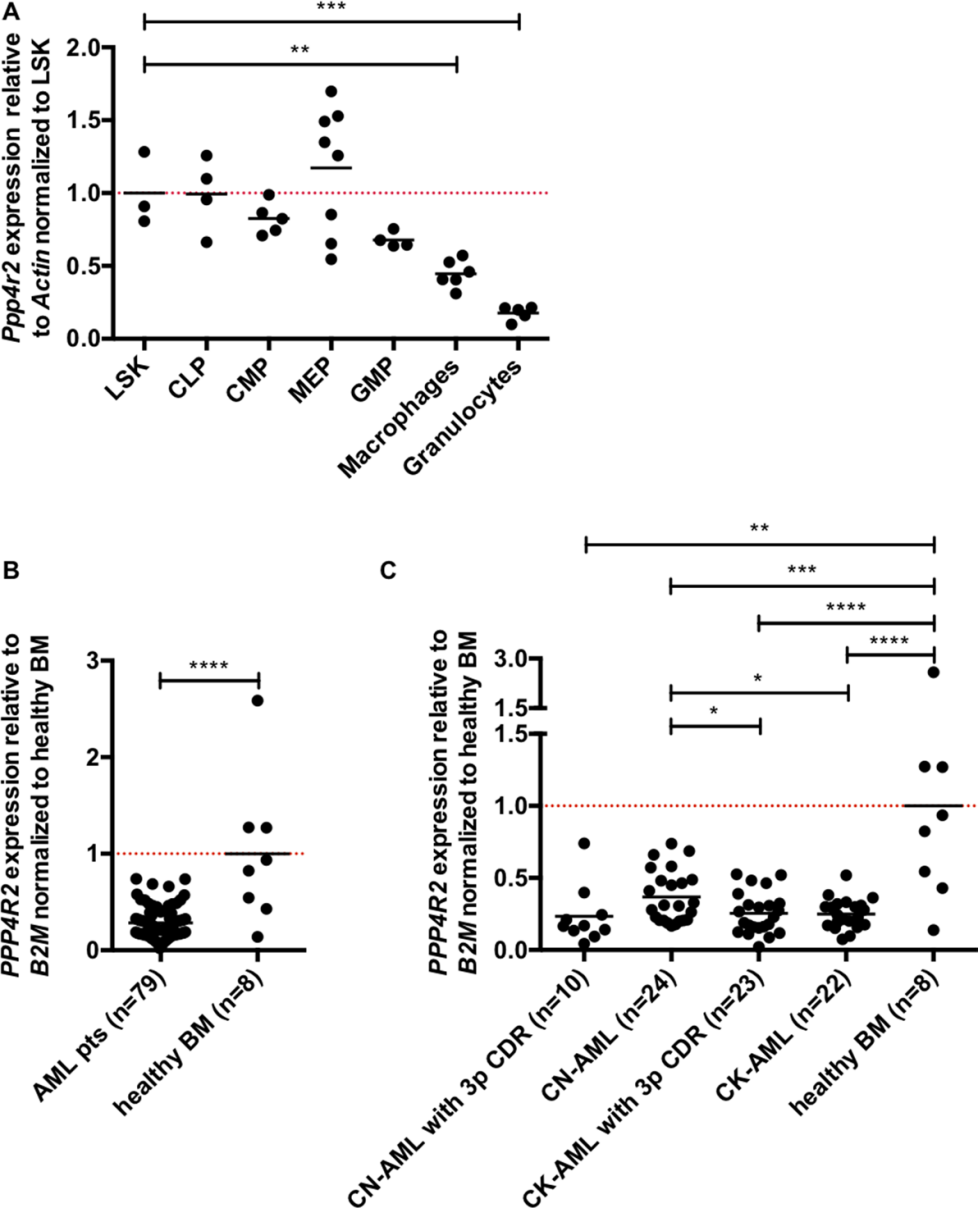
*Ppp4r2* is differentially expressed in hematopoiesis and neoplasia Expression of *Ppp4r2* in murine hematopoietic subpopulations and *PPP4R2* in AML patients was determined by qRT-PCR. (**A**) *Ppp4r2* single gene expression among murine hematopoietic subpopulations relative to the housekeeping gene *Actin* and normalized to Lin^-^/Sca1^+^/ckit^+^ (LSK) cells [LSK (*n* = 3); common lymphoid progenitors (CLP, *n* = 4); common myeloid progenitors (CMP, *n* = 5); megakaryocyte/erythrocyte progenitors (MEP, *n* = 8); granulocyte/monocyte progenitors (GMP, *n* = 4); macrophages (*n* = 6); granulocytes (*n* = 5)]. (**B**) *PPP4R2* mRNA expression relative to the housekeeping gene *beta-2 microglobulin* (*B2M*) in a selected cohort of AML patients (*n* = 79) normalized to healthy bone marrow (BM; *n* = 8). (**C**) *PPP4R2* mRNA expression relative to *B2M* in distinct AML subgroups [CN-AML with 3p CDR (*n* = 10), CN-AML (*n* = 24), CK-AML with 3p CDR (*n* = 23), CK-AML (*n* = 22)] normalized to healthy BM (*n* = 8). Data are represented by each individual data point and the mean. Statistical analyses were carried out using unpaired two-tailed *t*-test. A *p*-value ≤0.05 was considered significant, *p ≤ 0.05, **p ≤ 0.01, ***p ≤ 0.001, ****p ≤ 0.0001.

We next investigated *PPP4R2* mRNA expression levels during maturation of murine hematopoiesis as well as in primary human AML samples by quantitative real-time PCR (qRT-PCR). Single gene expression was analyzed in the following murine hematopoietic subpopulations: Lin^-^Sca^+^cKit^+^ cells (LSK), common myeloid progenitors (CMP), common lymphoid progenitors (CLP), megakaryocytic erythroid progenitors (MEP), granulocyte monocyte progenitors (GMP), macrophages, and granulocytes. Compared to LSK cells that comprise hematopoietic stem and progenitor cells (HSPC), *Ppp4r2* expression decreases significantly during myeloid differentiation (Figure 1A). We detected similar *Ppp4r2* mRNA levels in LSK and CLP, but decreased *Ppp4r2* expression in CMP and GMP and even lower expression in macrophages (*p* = 0.002), and granulocytes (*p* = 0.0003). Of note, publicly available microarray datasets [29] displayed comparable expression pattern of *PPP4R2* in normal human myelopoiesis with higher *PPP4R2* expression in HSPC, and lower expression in more mature myeloid cells (Supplementary Figure 1).

To obtain first insights in the role of *PPP4R2* in human leukemia, we investigated *PPP4R2* mRNA expression levels in a selected cohort of AML patients with and without 3p CDR [*n* = 79; 3p CDR within CN-AML (*n* = 10), CN-AML no 3p deletion (*n* = 24), 3p CDR within CK-AML (*n* = 23), CK-AML no 3p deletion (*n* = 22)] in comparison to bone marrow (BM) of healthy controls (*n* = 8). In general, *PPP4R2* was lower expressed in AML patients compared to healthy BM (*p <* 0.0001; Figure 1B). With regard to distinct subgroups compared to healthy BM, patients with 3p CDR within CN-AML (*p* = 0.007), 3p CDR within CK-AML (*p <* 0.0001) as well as CN-AML (*p* = 0.0005) and CK-AML (*p <* 0.0001) displayed significantly lower *PPP4R2* mRNA levels (Figure 1C). Compared to CN-AML, *PPP4R2* expression was significantly lower in the other subgroups 3p CDR within CN-AML (*p* = 0.07), 3p CDR within CK-AML (*p* = 0.02) as well as CK-AML (*p* = 0.01).

### *Ppp4r2* suppression regulates DNA damage response in normal murine BM cells

**Figure 2:**
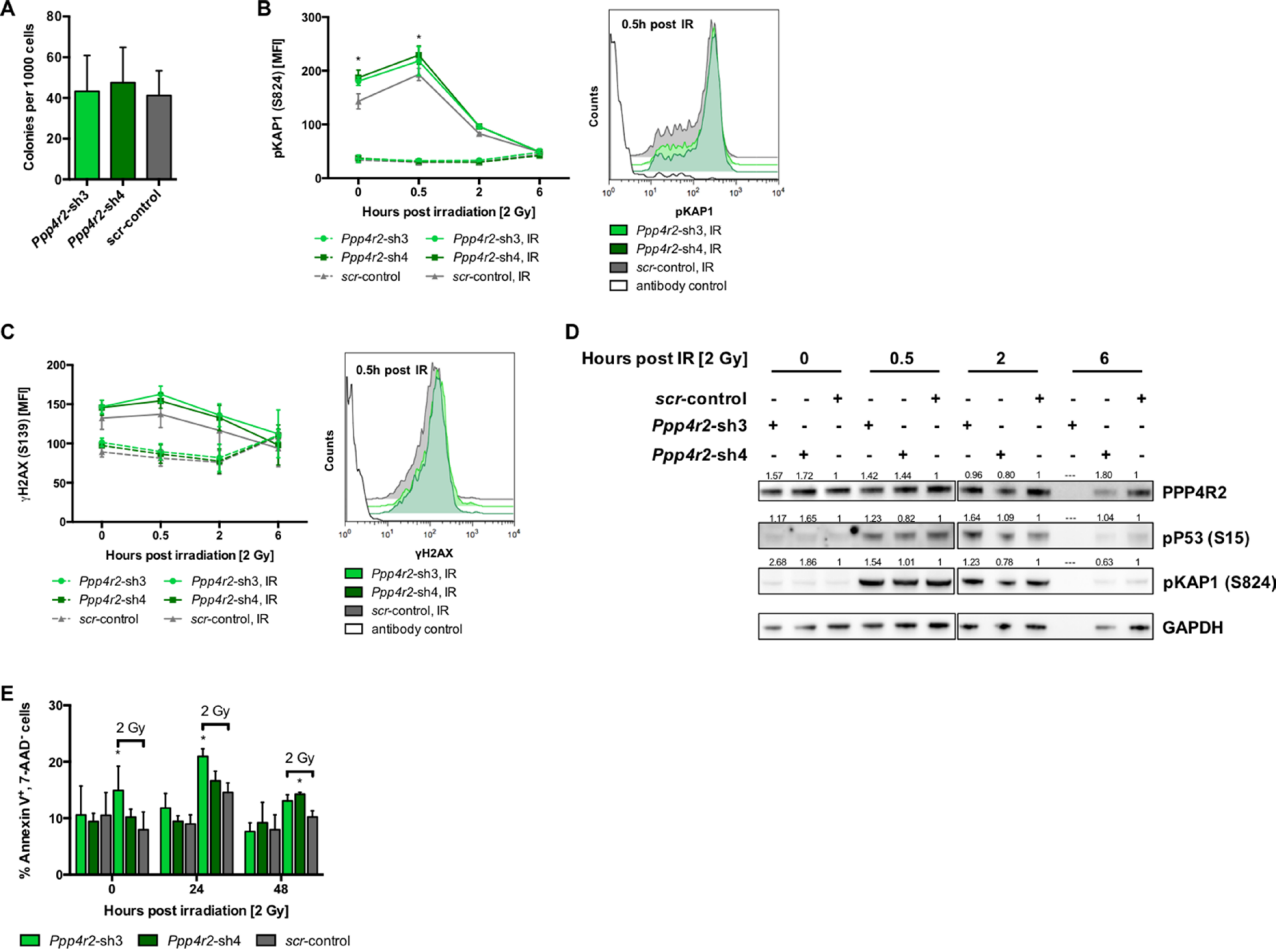
*Ppp4r2* suppression regulates DNA damage response in normal murine hematopoietic cells (**A**) Impact of *Ppp4r2* knockdown in murine Lin^-^ bone marrow (BM) cells on clonogenic growth determined by colony forming cells in methylcellulose (CFC-Assay; *n* = 4). (**B**) Impact of *Ppp4r2* knockdown in murine Lin^-^ BM cells on DNA damage response upon ionizing radiation (IR) with 2 Gy determined by the mean fluorescence intensity (MFI) of phosphorylated KRAB-domain associated protein 1 [pKAP1(S824)] at the indicated time post IR (*n* = 2). Representative histogram depicts the MFI shift of pKAP1 (S824) at 0.5 h post IR in either murine Lin^-^ BM cells with *Ppp4r2* knockdown or control. (**C**) DNA damage at indicated time post IR with 2 Gy in murine Lin^-^ BM cells upon *Ppp4r2* knockdown determined by the MFI of phosphorylated histone variant H2AX [γH2AX (S139); *n* = 3]. Representative histogram depicts the MFI shift of γH2AX (S139) at 0.5 h post IR in murine Lin^-^ BM cells with either *Ppp4r2* knockdown or control. (**D**) Representative Western Blot displaying the effect of IR on phosphorylation of KAP1 (S824) and P53 (S15) in Lin^-^ BM cells with either *Ppp4r2* knockdown or control. Vertical lines have been inserted to indicate a repositioned gel lane. (**E**) Apoptosis induction upon IR with 2 Gy displayed as the percentage of AnnexinV^+^/7AAD^-^ Lin^-^ BM cells with either *Ppp4r2* knockdown or control at the indicated time post IR (*n* = 3). Data are represented by the mean ± SD. Statistical analyses were carried out using unpaired two-tailed *t*-test or multiple *t*-tests corrected for multiple comparisons using the Holm-Sidak method. A *p*-value ≤ 0.05 was considered significant, *p ≤ 0.05.

Differential *PPP4R2* expression in hematopoietic cells and primary AML samples prompted us to hypothesize that *PPP4R2* might play a functional role in leukemia. We genetically inactivated *Ppp4r2* by RNAi in murine hematopoietic progenitor cells using two different shRNAs (*Ppp4r2*-sh3, *Ppp4r2*-sh4) and a non-targeting control (Supplementary Figure 2A). First, we assessed whether *Ppp4r2* impacts on the ability of HSPC to divide and proliferate to form colonies and cell clusters. There was no apparent effect of *Ppp4r2* knockdown on colony formation of murine hematopoietic progenitor cells (Figure 2A). PPP4R2 has been shown to be involved in DNA repair by governing the de-phosphorylation activity and substrate specificity of the PPP4 complex [23, 24, 26, 27]. To investigate the contribution of PPP4R2 to DNA repair of normal hematopoietic cells, we assessed for DDR signaling proteins pKAP1 (S824) and γH2AX (S139) by flow cytometry. Determination of DDR induced by IR with 2 Gy [directly after irradiation (further referred to as 0 h post IR), 0.5 h, 2 h, and 6 h post IR] showed that *Ppp4r2* knockdown resulted in elevated levels of pKAP1 (*Ppp4r2*-sh4: 0 h post IR, *p* = 0.003; 0.5 h post IR, *p* = 0.008), most prominent at 0.5 h post IR as depicted in Figure 2B. DNA damage measured by γH2AX was increased upon *Ppp4r2* knockdown followed by IR with 2 Gy (Figure 2C). Western Blot analysis confirmed elevated pKAP1 protein levels upon knockdown of *Ppp4r2* as well as activated P53 [phosphorylated P53 (S15), pP53] after IR with 2 Gy (Figure 2D). Further investigation of apoptosis induction in response to DNA damage determined as percentage of AnnexinV^+^/7-AAD^-^ cells displayed an increase in early apoptotic cells upon *Ppp4r2* knockdown (*Ppp4r2*-sh3: 0 h post IR, *p* = 0.004; 24 h post IR, *p* = 0.007; *Ppp4r2*-sh4: 48 h post IR, *p* = 0.02; Figure 2E).

### *Ppp4r2* loss-of-function enhances DNA damage in murine leukemic BM cells

**Figure 3:**
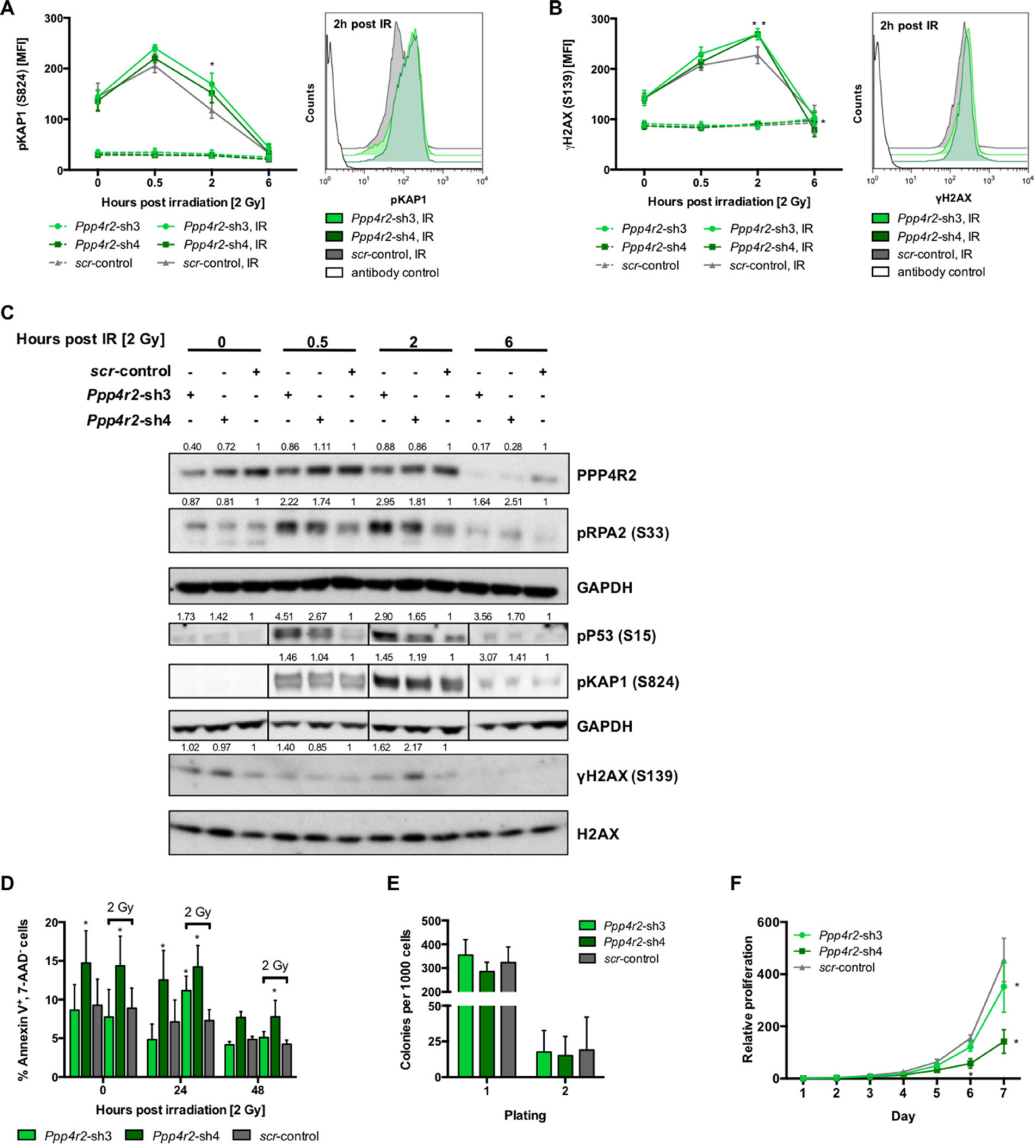
*Ppp4r2* loss-of-function enhances DNA damage in murine leukemic bone marrow cells (**A**) Impact of *Ppp4r2* knockdown in murine *MLLT3-KMT2A* transformed Lin^-^ bone marrow (BM) cells on DNA damage response upon ionizing radiation (IR) with 2 Gy measured by the mean fluorescence intensity (MFI) of phosphorylated KRAB-domain associated protein 1 [pKAP1 (S824)] at the indicated time post IR (*n* = 3). Representative histogram depicts the MFI shift of pKAP1 (S824) at 2 h post IR in either murine *MLLT3-KMT2A* transformed Lin^-^ BM cells with *Ppp4r2* knockdown or control. (**B**) DNA damage at indicated time post IR with 2 Gy in murine *MLLT3-KMT2A* Lin^-^ BM cells upon *Ppp4r2* knockdown determined by the MFI of phosphorylated histone variant H2AX [γH2AX (S139); *n* = 3]. Representative histogram depicts the MFI shift of γH2AX at 2 h post IR in murine *MLLT3-KMT2A* transformed Lin^-^ BM cells with either *Ppp4r2* knockdown or control. (**C**) Representative Western Blot displaying the effect of IR on phosphorylation of the key DDR protein RPA2 (S33), P53 (S15), KAP1 (S824), and H2AX (S139) in murine *MLLT3-KMT2A* Lin^-^ BM cells with either *Ppp4r2* knockdown or control. Vertical lines have been inserted to indicate a repositioned gel lane. (**D**) Apoptosis induction upon IR with 2 Gy displayed as the percentage of AnnexinV^+^/7AAD^-^ murine *MLLT3-KMT2A* Lin^-^ BM cells with either *Ppp4r2* knockdown or control at the indicated time post IR (*n* = 5). Impact of *Ppp4r2* knockdown in murine *MLLT3-KMT2A* transformed Lin^-^ BM cells on (**E**) clonogenic growth and replating capacity determined by colony forming cells in methylcellulose (CFC-Assay; *n* = 5), and (**F**) proliferation potential measured by MTS-Assay (*n* = 3). Data are represented by the mean ± SD. Statistical analyses were carried out using unpaired two-tailed *t*-test or multiple *t*-tests corrected for multiple comparisons using the Holm-Sidak method. A *p*-value ≤ 0.05 was considered significant, *p ≤ 0.05.

To determine effects of *Ppp4r2* loss-of-function in a leukemic state, *Ppp4r2* knockdown experiments were carried out in murine *myeloid/lymphoid or mixed-lineage leukemia translocated to 3 -* *lysine methyltransferase 2A* (*MLLT3*-*KMT2A*, formerly known as *MLL-AF9*) transformed Lin^-^ BM cells, which fully recapitulate human myeloid *KMT2A* leukemia [30–32]. We genetically inactivated *Ppp4r2* in murine *MLLT3-KMT2A* leukemic cells by lentiviral transduction with two shRNAs (*Ppp4r2*-sh3, *Ppp4r2*-sh4) targeting *Ppp4r2* (Supplementary Figure 2B). Additionally, qRT-PCR analyses of the other PPP4 regulatory subunits *Ppp4r1*, *Ppp4r3a*, and *Ppp4r3b* were performed to validate specific knockdown of *Ppp4r2* in the *MLLT3-KMT2A* leukemia model (Supplementary Figure 2C). To evaluate DNA damage and response to DSB, cells with either *Ppp4r2* knockdown or control were exposed to IR with 2 Gy and DDR signaling proteins pKAP1 (S824) and γH2AX (S139) were determined by flow cytometry at 0 h, 0.5 h, 2 h, and 6 h post IR. DDR upon *Ppp4r2* knockdown led to the accumulation of pKAP1 (*Ppp4r2*-sh3: 2 h post IR, *p* = 0.002; Figure 3A). DNA damage assessed by γH2AX showed elevated levels upon *Ppp4r2* knockdown followed by IR (*Ppp4r2*-sh3: 2 h post IR, *p* = 0.002; *Ppp4r2*-sh4: 2 h post IR, *p* = 0.0009; 6 h post IR, *p* = 0.01; Figure 3B). Western Blot analyses displaying the phosphodynamics of key DDR proteins after IR with 2 Gy confirmed elevated pKAP1 and γH2AX protein levels in murine *MLLT3-KMT2A* leukemic cells with *Ppp4r2* knockdown. In addition, they further revealed activation of P53 by phosphorylation as well as RPA2 hyperphosphorylation upon knockdown of *Ppp4r2* in murine *MLLT3-KMT2A* leukemic cells (Figure 3C). Determination of early apoptotic cells in response to IR after 0 h, 24 h, and 48 h revealed increased sensitivity to IR upon *Ppp4r2* knockdown displaying higher numbers of early apoptotic cells represented by the percentage of AnnexinV^+^/7-AAD^-^ cells (*Ppp4r2*-sh3: 24 h post IR, *p* = 0.007; *Ppp4r2*-sh4: 0 h no IR, *p* = 0.007; 24 h no IR, *p* = 0.008; 0 h post IR, *p* = 0.002; 24 h post IR, *p* = 0.0002; 48 h post IR, *p* = 0.03; Figure 3D). Colony formation ability of *MLLT3-KMT2A* transformed leukemic cells was not affected upon *Ppp4r2* suppression (Figure 3E). Proliferative capacity was further investigated using MTS-Assay, which revealed reduced metabolic activity upon *Ppp4r2* knockdown in murine *MLLT3-KMT2A* transformed leukemic cells (*Ppp4r2*-sh3: day 7, *p* = 0.001; *Ppp4r2*-sh4: day 7, *p <* 0.0001; Figure 3F).

### Re-expression of *PPP4R2* in 3p deleted human leukemic cells restores DNA repair upon irradiation

**Figure 4:**
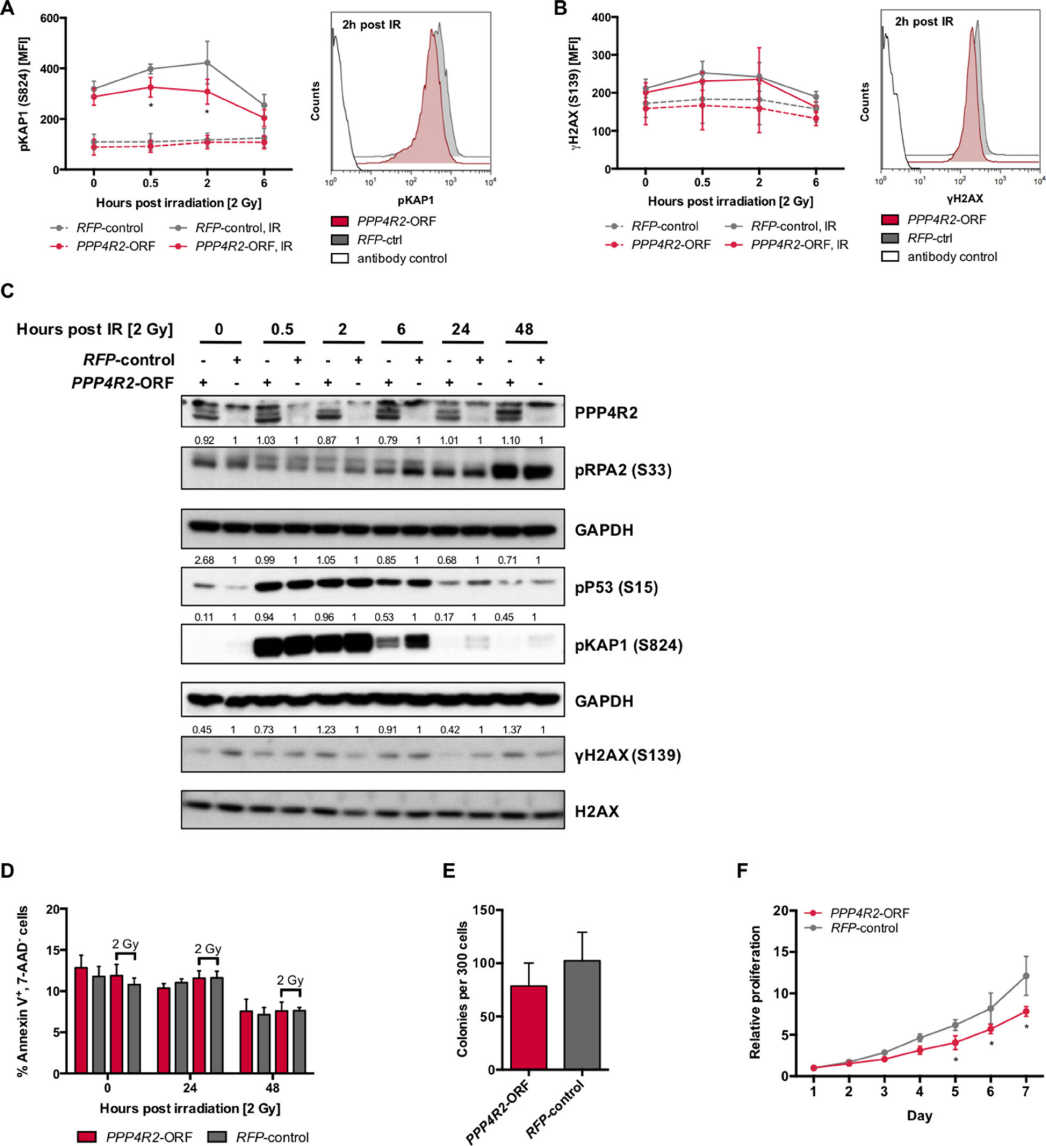
Re-expression of *PPP4R2* restores DNA repair in leukemic cells with 3p microdeletion (**A**) Impact of *PPP4R2* restoration in human leukemic MEG-01 cells on DNA damage response upon ionizing radiation (IR) with 2 Gy determined by the mean fluorescence intensity (MFI) of phosphorylated KRAB-domain associated protein 1 [pKAP1 (S824)] at the indicated time post IR (*n* = 5). Representative histogram depicts the MFI shift of pKAP1 (S824) at 2 h post IR in either MEG-01 cells with *PPP4R2* restoration or control. (**B**) DNA damage at indicated time post IR with 2 Gy in MEG-01 cells upon *PPP4R2* re-expression determined by the MFI of phosphorylated histone variant H2AX [γH2AX (S139); *n* = 4]. Representative histogram depicts the MFI shift of γH2AX (S139) at 2 h post IR in MEG-01 cells with either *PPP4R2* restoration or control. (**C**) Representative Western Blot displaying the effect of IR with 2 Gy on phosphorylation of the DDR protein RPA2 (S33), P53 (S15), KAP1 (S824), and H2AX (S139) in MEG-01 cells with either *PPP4R2* restoration or control. (**D**) Effect of *PPP4R2* re-expression and exposure to IR with 2 Gy on apoptosis induction displayed as the percentage of AnnexinV^+^/7AAD^-^ cells in comparison to control (*n* = 4). Impact of *PPP4R2* restoration on (**E**) clonogenic growth determined by colony forming cells in methylcellulose (CFC-Assay; *n* = 4), and (**F**) proliferation potential measured by MTS-Assay (*n* = 3). Data are represented by the mean ± SD. Statistical analyses were carried out using unpaired two-tailed *t*-test or multiple *t*-tests corrected for multiple comparisons using the Holm-Sidak method. A *p*-value ≤ 0.05 was considered significant, *p ≤ 0.05.

Next, we performed reconstitution experiments in the *PPP4R2*-deficient human leukemic cell line MEG-01 by lentiviral transduction of the *PPP4R2* open reading frame (*PPP4R2*-ORF; Supplementary Figure 2D) to evaluate the role of *PPP4R2* in hematologic neoplasia associated with 3p loss. DNA damage was induced by IR followed by investigation of DDR proteins pKAP1 (S824) and γH2AX (S139) by flow cytometry at 0 h, 0.5 h, 2 h, and 6 h post IR. In comparison to irradiated MEG-01 cells used as control, re-expression of *PPP4R2* resulted in significantly lower pKAP1 levels in response to induction of DSB by IR (0.5 h post IR, *p* = 0.01; 2 h post IR, *p* = 0.0001), which is represented by the shift to the left of pKAP1 MFI at 2 h post IR (Figure 4A). DNA damage determined by γH2AX after irradiation showed less phosphorylation in *PPP4R2* expressing MEG-01 cell line, more clearly represented by the respective histogram showing a MFI shift of γH2AX to the left (Figure 4B). Western Blot analysis confirmed reduced pKAP1 protein in MEG-01 cells upon re-expression of *PPP4R2* and exposure to IR (Figure 4C). Additionally, we observed reduced phosphorylation of the DDR proteins pP53, γH2AX, and pRPA2 in comparison to MEG-01 control (Figure 4C). Although we did not observe induction of apoptosis upon DNA damage, independent of *PPP4R2* expression (Figure 4D), re-expression of *PPP4R2* impaired colony forming potential compared to control (*p* = 0.07; Figure 4E). Determination of cell proliferation and viability by metabolic activity with MTS-Assay showed reduced proliferation of the human myeloid leukemic cell line MEG-01 after re-expression of *PPP4R2* (day 7, *p* < 0.0001; Figure 4F), which was also confirmed by counting viable cells by trypan blue exclusion method (data not shown).

### Gene expression profiling (GEP) and whole exome sequencing (WES) of primary AML samples identified enriched gene sets associated with DNA repair

**Figure 5:**
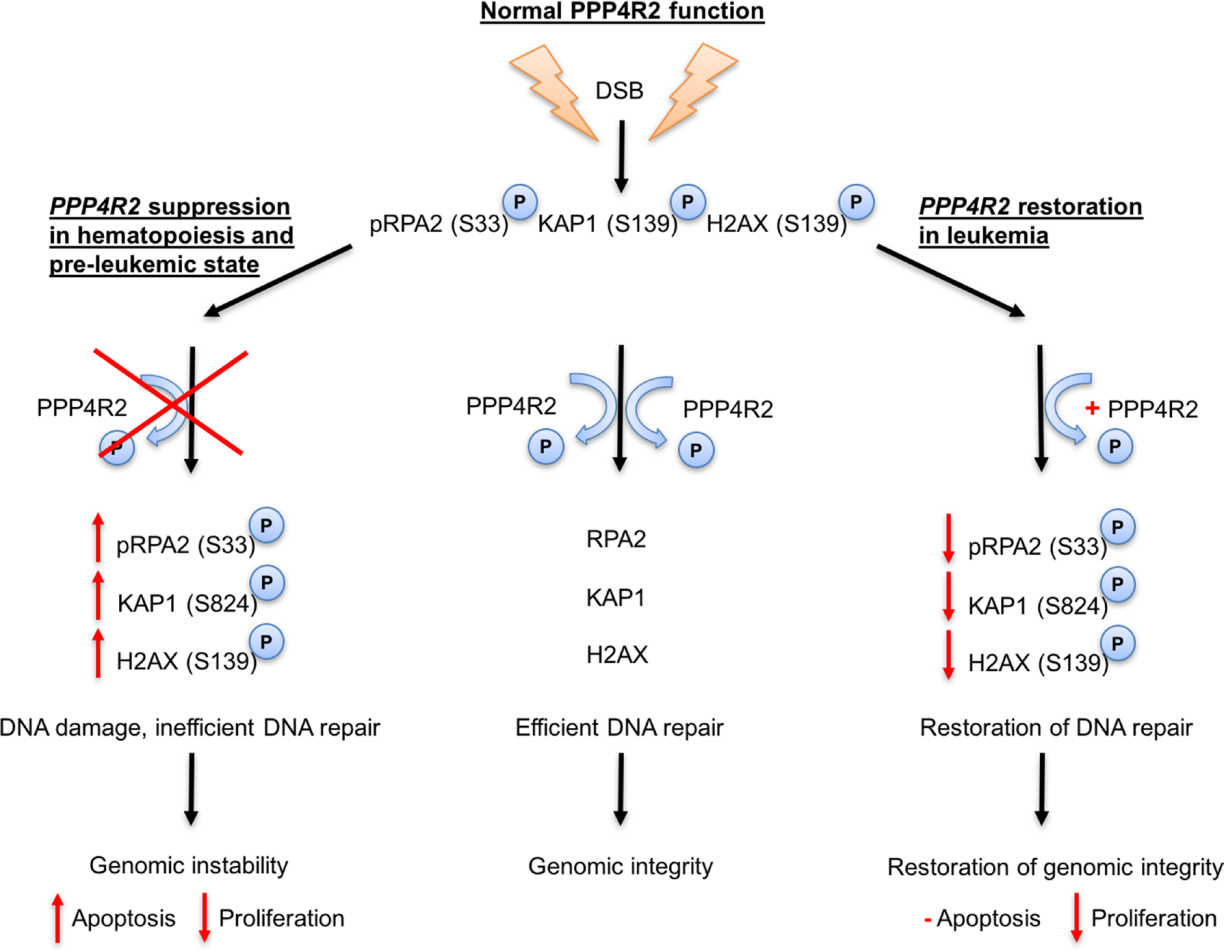
Proposed model for PPP4R2 and its involvement in DNA damage response in normal hematopoietic and leukemic cells

Based on our findings that *PPP4R2* is differentially expressed in hematopoiesis and leukemia as well as its involvement in DDR (Figure 5), we aimed to investigate whether our hypothesis that deregulated DNA repair promoted by *PPP4R2* depletion may contribute to leukemia development is also reflected by global gene expression data of primary AML samples.

Therefore, we performed a supervised analysis determining *PPP4R2* co-expressed genes by comparing 10% of AML cases with the highest and lowest *PPP4R2* expression. This analysis revealed 5463 significantly differentially expressed genes at a false discovery rate below 1%. Notably, the top 1000 most significant genes of this signature were significantly enriched for members of pathways associated with cancer [e.g. *ABL proto-oncogene* *1* (*ABL1*)*, mutS homolog 6* (*MSH6*)*, nucleoporin 98* (*NUP98*)*, tumor protein 53 binding protein 2* (*TP53BP2*)] and DNA repair [e.g. *breast cancer 1* (*BRCA1*)*, MSH6, TP53BP2, small ubiquitin-like modifier 1* (*SUMO1*)] as well as apoptosis following DNA damage [e.g. *protein tyrosine phosphatase non-receptor type 12* (*PTPN12*)*, nuclear factor kappa B subunit 1* (*NFKB1*)] (Supplementary Table 3). In accordance, an analysis comparing CN-AML cases with 3p CDR (*n* = 9) with CN-AML without 3p deletion (*n* = 161) revealed a significant enrichment of gene sets associated with cancer, DNA repair as well as cell death and survival, which further supports a strong impact of *PPP4R2* in 3p deleted AML (Supplementary Table 4).

Next, we were interested whether primary AML patients with 3p microdeletion also harbor a distinct gene mutation pattern that might further support the deregulation of the respective pathways. Whole exome sequencing of five paired diagnosis/remission samples from CN-AML cases with 3p CDR showed on average 14 tumor-specific mutations per AML (Table 1). Beside significant enrichment for hits and pathways typically associated with AML [e.g. *nucleophosmin* (*NPM1*), *fms related tyrosine kinase 3* (*FLT3*), *NRAS proto-oncogene GTPase* (*NRAS*)], we also found a notable number of hits in pathways associated with response to DNA damage as well as apoptosis and cell survival [e.g. *mutS homolog 2* (*MSH2*)*, forkhead box O3* (*FOXO3*)*, cell division cycle 27* (*CDC27*)] (Supplementary Table 5).

**Table 1:** Mutations detected by whole exome sequencing of AML cases with 3p microdeletion (*n* = 5)

Patient	Tumor-specific mutations (somatic)	Pre-leukemic mutations (germline/remission)
1	*TEX11, GATA2*^a^*, ZNF687, CCT7, CATSPER4, NRAS*^a^*,* ***NPM1***^a^*, TLR4*^a^*, SYNE2, POSTN*^a^*, CTDSP2, GXYLT1*^a^*, GXYLT1*^a^*, CTBP2*^a^	***DNMT3A***^a^
2	*CACNA1A, BMP5*^a^*, TRPC6, CYFIP2*^a^*, RBX1, PRLR, IRF2BP2, UNC13C*^a^*, ARMC9, SENP3,* ***NPM1***^a^	*RUNX1*^a^*,* ***DNMT3A***^a^
3	*ADAMTS12, DISP1, KIAA1551, OR5H1, DNAH9, TJP1, PLPPR1, RIT1*^a^*,* ***NPM1***^a^*, HSPBAP1, CCDC178*	*NPAT,* ***DNMT3A***^a^
4	***DNMT3A***^a^*, RBM46, PHGR1, APOA5, HAUS1, ZNF469, CHFR, MSH2, SLC12A7, KAT6B, FAM184A, PCDH15, AGAP1, PFN3,* ***NPM1***^a^*, ADGRA3*^a^*, FOXO3, FLT3*	
5	*HIPK3, PKP1, CDC42EP1, CMTM3*^a^*, SPDYC, WT1*^a^*, OSMR, MYT1L*^a^*, FCN1, ACLY, COL19A1, DMBT1, CDC27,* ***NPM1***^a^*, CTNNA3*	

^a^mutated in cancer according to the Catalogue of Somatic Mutations in Cancer (COSMIC).

Genes in bold are commonly mutated.

TEX11, testis expressed gene 11; GATA2, GATA binding protein 2; ZNF687, zinc finger protein 687; CCT7, chaperon containing TCP1 subunit 7; CATSPER4, cation channel sperm associate 4; NRAS, NRAS proto-oncogene GTPase; NPM1, nucleophosmin; TLR4, toll like receptor 4; SYNE2, spectrin repeat containing nuclear envelope protein 2; POSTN, periostin; CTDSP2, CTD small phosphatase 2; GXYLT1, glucoside xylosyltransferase 1; CTBP2, C-terminal binding protein 2; CACNA1A, calcium voltage-gated channel subunit alpha1 A; BMP5, bone morphogenetic protein 5; TRPC6, transient receptor potential cation channel subfamily C member 6; CYFIP2, cytoplasmic FMR1 interacting protein 2; RBX1, ring-box 1; PRLR, prolactin receptor; IRF2BP2, interferon regulatory factor 2 binding protein 2; UNC13C, unc-13 homolog C; ARMC9, armadillo repeat containing 9; SENP3, SUMO1/sentrin/SMT3 specific peptidase 3; ADAMTS12, ADAM metallopeptidase with thrombospondin type 1 motif 12; DISP1, dispatched RND transporter family member 1; OR5H1, olfactory receptor family 5 subfamily H member 1; DNAH9, dynein axonemal heavy chain 9; TJP1, tight junction protein 1; PLPPR1, phospholipid phosphatase related 1; RIT1, Ras like without CAAX1; HSPBAP1, HSPB1 associated protein 1; CCDC178, coiled-coil domain containing 178; DNMT3A, DNA methyltransferase 3 alpha; RBM46, RNA binding motif protein 46; PHGR1, proline, histidine and glycine rich 1; APOA5, apolipoprotein A5; HAUS1, HAUS augmin like complex subunit 1; ZNF469, zinc finger protein 469; CHFR, checkpoint with forkhead and ring finger domains; MSH2, mutS homolog 2; SLC12A7, solute carrier family 12 member 7; KAT6B, lysine acetyltransferase 6B; FAM184A, family with sequence similarity 184 member A; PCDH15, protocadherin related 15; AGAP1, ArfGAP with GTPase domain, ankyrin repeat and PH domain 1; PFN3, profilin 3; ADGRA3, adhesion G protein-coupled receptor A3; FOXO3, forkhead box O3; FLT3, fms related tyrosine kinase 3; HIPK3, homeodomain interacting protein kinase 3; PKP1, plakophilin 1; CDC42EP1, CDC42 effector protein 1; CMTM3, CKLF like MARVEL transmembrane domain containing 3; SPDYC, speedy/RINGO cell cycle regulator family member C; WT1, Wilms tumor 1; OSMR, oncostatin M receptor; MYT1L, myelin transcription factor 1 like; FCN1, ficolin 1; ACLY, ATP citrate lyase; COL19A1, collagen type XIX alpha 1 chain; DMBT1, deleted in malignant brain tumors 1; CDC27, cell division cycle 27; CTNNA3, catenin alpha 3; RUNX1, runt related transcription factor 1; NPAT, nuclear protein, coactivator of histone transcription.

Finally, we performed a comprehensive analysis of the gene expression profiles and mutational patterns of AML patients obtained by GEP and WES analyses to identify common deregulated genes and functional-related pathways. To this end, we determined overlapping differentially expressed and mutated genes or enriched gene sets obtained by comparison of analyses of 1) AML with high *versus* low global *PPP4R2* expression, 2) CN-AML with 3p microdeletion *versus* CN-AML without 3p microdeletion, and 3) mutated genes of AML patients with 3p microdeletion (Supplementary Figure 3). Mutated or differentially expressed *DNA methyltransferase 3 alpha* (*DNMT3A*) was shared among all three types of analyses. Common leukemia-associated genes have been identified by the investigation of gene sets obtained by comparison of analysis 1 with analysis 3 [*FLT3* and *C-terminal binding protein 2* (*CTBP2*)] and analysis 2 with analysis 3 [*NRAS* and *GATA binding protein 2* (*GATA2*)]. The comparison of analysis 1 to analysis 2 identified 98 common deregulated genes. Of note, the comparison of enriched gene sets among all three analyses identified one common deregulated gene set (DACOSTA_UV_RESPONSE_VIA_ERCC3_DN). This gene set comprises genes that were downregulated in fibroblasts expressing mutant forms of *ERCC excision repair 3* (*ERCC3*) after ultraviolet irradiation, representing a transcriptional profile that was specifically regulated upon ultraviolet irradiation depending on the DNA repair capacity of the cell [33].

## DISCUSSION

We previously identified a recurrent microdeletion at chromosome 3p14.1-p13 in AML [7–9] that was also described in prostate and cervical cancer, and was supposed to harbor several potential cooperative tumor suppressor genes [10, 11]. In the present work we report on the functional characterization of *PPP4R2*, one candidate gene located in the CDR, in the context of hematopoiesis and leukemia.

Mutation analyses of primary AML patient samples revealed that the function of *PPP4R2* is not affected by mutations in AML. This is in line with public sequencing data from The Cancer Genome Atlas (TCGA), which showed neither mutation nor amplification of *PPP4R2* but deletion in 1.6% of AML cases [3]. This finding indicates that *PPP4R2* function might rather be affected by gene deletions or epigenetic deregulation than gene mutations.

Our data concerning *PPP4R2* expression levels suggest a functional relevance of *PPP4R2* in HSPC and might indicate its requirement in normal hematopoietic cells during early myelopoiesis. Publicly available microarray data provide evidence for such an expression pattern also in human myelopoiesis [29]. The functional properties of PPP4R2 are not thoroughly investigated so far, thus we also interpreted the results of our study with respect to the catalytic subunit of PPP4, whose activity is controlled by regulatory subunits like PPP4R2. Besides embryonic lethality of *Ppp4*-null mice, PPP4 has been shown to be essential for development of hematopoietic cells. T-/B-cell specific *Ppp4* deletion led to an early block in T-/B-cell development and abnormal thymocyte maturation [18–20]. Furthermore, *PPP4R2* expression has been associated with differentiation in neuronal NSC-34 cells, in which *PPP4R2* depletion resulted in abnormal and strongly inhibited differentiation towards differentiation block [21]. Based on our data decreased expression in primary AML cases may contribute to leukemia initiation or development. Loss of *PPP4R2* expression in CK-AML, an AML subgroup associated with substantial genomic instability and unfavorable prognosis, might point to AML cells that select for *PPP4R2* depletion along with perturbed maintenance of genomic integrity. Recent expression analyses of primary lung cancer cases revealed lower *PPP4R2* levels in late stages of tumor progression characterized by lymph node metastases than in matched primary lung cancer tissue [22]. Genetic inactivation of *PPP4R2* in the lung cancer cell line A549 displayed significant more metastatic progression in mice, indicating that lower levels of *PPP4R2* support metastatic processes e.g. migration and invasion to promote tumor progression.

Our functional studies implicate that *Ppp4r2* contributes to DNA repair of normal murine HSPC by positive regulation of the PPP4 de-phosphorylation activity of DDR signaling proteins as well as induction of apoptosis. Clonogenicity was not apparently affected upon suppression of *Ppp4r2* in normal murine Lin^-^ BM cells. Comparable results have been described for PPP4 in the context of lymphopoiesis. Loss of *Ppp4* in B-cells of conditional knockout mice was associated with impaired cell development, increased apoptosis, and persistent DNA damage upon immunoglobulin recombination that is typically repaired by NHEJ [19, 20]. Interestingly, downregulation of *PPP4* and its regulatory subunits in non-malignant human embryonic kidney cell line reduced HR- and NHEJ-mediated DNA repair and resulted in increased DNA damage [23, 27].

Our report further provides evidence for *Ppp4r2* affecting DNA repair of leukemic hematopoietic cells. Response to DNA damage upon *Ppp4r2* knockdown in the murine *MLLT3-KMT2A* transformed leukemia model was accompanied by enhanced DNA damage and/or impaired DNA repair. Previous reports have shown that *PPP4R2* depletion in MCF-7 breast cancer cells caused increased KAP1 phosphorylation in response to camptothecin- or etoposide-mediated DNA damage, and was associated with a significant reduction of NHEJ-mediated DNA repair [27]. Phosphorylation of KAP1 caused global decondensation of chromatin that might increase susceptibility for DNA damage [34], and transcriptional de-repression of e.g. p21 and P53 target genes [35, 36]. Moreover, γH2AX as a readout for DNA damage has also been shown to be controlled by *PPP4R2* in osteosarcoma U2OS or cervix carcinoma HeLa cell line, even independent of exogenous DNA damage [23, 24]. Depletion of *PPP4R2* or the catalytic subunit of PPP4 delayed γH2AX de-phosphorylation during recovery from irradiation [24]. *PPP4* deficiency particularly impeded HR-mediated repair of DSB by unresolved γH2AX during replication [23]. Although Nakada *et al*. proposed that de-phosphorylation of γH2AX upon DNA damage by irradiation is rather a function attributed to PPP4, other PP2A phosphatases also target γH2AX to facilitate DSB repair and might compensate the effect of *Ppp4r2* knockdown [24, 37]. Furthermore, the main PPP4 complex that de-phosphorylates pKAP1 or γH2AX includes the PPP4R3β subunit, which could explain mitigated effects in response to DNA damage seen in our study upon genetic inactivation of *Ppp4r2* [23, 24, 26]. Knockdown of *Ppp4r2* in murine *MLLT3-KMT2A* leukemia followed by induction of DSB was further accompanied by elevated levels of pRPA2, which is a specific and relevant substrate of the PPP4R2-containing PPP4 complex that impaired HR-mediated DSB repair by inefficient DDR [25]. Hence, suppression of *Ppp4r2* in the murine *MLLT3-KMT2A* leukemia model reduced the de-phosphorylation of key DDR proteins, which resulted in elevated DDR presenting inefficient DNA repair followed by accumulation of DNA damage. Intriguingly, downregulation of the catalytic subunit of the PPP4 complex in human T-cell leukemic cell lines Jurkat and CEM-C7 by RNAi displayed substantially increased mutation frequency upon ultraviolet irradiation, supporting an important role of the PPP4 complex in DNA repair of leukemic cells [38]. Impaired DNA repair by loss of *Ppp4r2* function potentially promotes genomic instability, leading to apoptosis induction and compromised proliferative capacity represented by lower metabolic activity. Colony formation capability of *MLLT3-KMT2A* transformed hematopoietic progenitors was not apparently affected by *Ppp4r2* knockdown. A more detailed examination of colonies could associate smaller colonies upon *Ppp4r2* knockdown with reduced growth rate. However, the impact of downregulated *PPP4* or its regulatory subunit *PPP4R2* on proliferation and apoptosis is controversially discussed. Comparable results were described upon *PPP4R2* knockdown in neuronal NSC-34 cells, which revealed increased apoptosis rate upon DNA damage by etoposide treatment [21]. *PPP4* depletion in murine thymocytes or HEK293T cells resulted in decreased proliferation and enhanced apoptosis [18, 39]. In contrast, downregulation of the catalytic subunit in T-leukemic cell lines, human peripheral blood T-lymphocytes, or HEK293T cells increased proliferation and inhibited apoptosis in response to different apoptotic stimuli [38, 40, 41]. Such cell type-dependent differences imply that the PPP4 complex needs to be tightly regulated. Altered *PPP4R2* expression might impact on multiple cellular functions such as cell survival, not at least due to its involvement in centrosome maturation and microtubule organization [39, 42].

Our reconstitution experiments indicate that re-expression of *PPP4R2* reduces DNA damage probably through improved DNA repair in leukemic cells. Restoration of DNA repair by *PPP4R2* expression in human myeloid leukemic cell line MEG-01 and additional deregulation of apoptosis pathways may account for lack of DNA damage triggered apoptosis. It is feasible that the involvement of the PPP4R2-containing PPP4 complex in centrosome maturation or spliceosome assembly is responsible for lower proliferation rate and colony formation upon ectopic expression of *PPP4R2* [39, 43]. This observation suggests that a certain level of PPP4R2 is relevant while decrease as well as increase of PPP4R2 perturbs cell homeostasis and viability. In osteosarcoma cell line U2OS it has been demonstrated that PPP4R2 in complex with the catalytic subunit was able to de-phosphorylate γH2AX in a dose-dependent manner [24]. In further accordance with our data, pKAP1 was described to be a substrate of the PPP4 complex, and overexpression of the catalytic subunit in breast cancer cell line MCF-7 resulted in decreased levels of camptothecin-induced pKAP1 [27]. The more specific de-phosphorylation activity of the PPP4R3β-containing PPP4 complex on pKAP1 and γH2AX could explain moderate effects upon ectopic *PPP4R2* expression in our study [23, 24, 26]. Moreover, highly proliferative and aggressive leukemic cells have to cope with DNA damage and activated DNA repair signaling. Additional genetic alterations in the *PPP4R2*-deficient human myeloid leukemic cell line MEG-01 permit survival of cells that acquired genomic instability providing an advantage to select for subclones, and indicating a cooperative role for *PPP4R2* deficiency in leukemia progression. In line with this, neuronal NSC-34 cells overexpressing *PPP4R2* were protected from apoptosis under basal conditions as well as in response to the DNA damaging agent etoposide [21].

GEP and WES data obtained from primary AML samples further support our model that *PPP4R2* is needed in immature cells to ensure genomic stability during the proliferative processes prior to cellular differentiation. Alterations in AML by either loss of *PPP4R2* (3p deletion) and/or downregulation of *PPP4R2* (possibly by epigenetic silencing) might enable the malignant cells to promote and rather tolerate genomic instability allowing faster cell turnover. Co-expressed genes and pathway enrichment analysis strongly highlight the involvement of *PPP4R2* in DNA repair mechanisms also in primary AML. In further accordance, GEP signatures and exome sequencing of leukemias with 3p microdeletion support the disturbance of the respective pathways by low *PPP4R2* expression, which might provide an advantage for subclones with 3p microdeletion. Despite co-occurrence of leukemia-associated differentially expressed and mutated genes, gene set enrichment analyses identified one common gene set comprising downregulated genes expressed in fibroblasts with mutant forms of *ERCC3* upon ultraviolet irradiation [33]. Intriguingly, mutations in *ERCC3*, a known cancer gene, can result in xeroderma pigmentosum with Cockayne syndrome or trichothiodystrophy, which are prominent diseases associated with defective DNA repair and predisposition to skin cancer [44–47].

In conclusion, we identified involvement of *PPP4R2* in DDR of both normal hematopoietic and leukemic cells via deregulation of the PPP4 complex. Impaired DNA repair and enhanced DNA damage by *PPP4R2* suppression might promote genomic instability, one possible mechanism by which 3p microdeletions could potentially contribute to the pathogenesis of AML. Intriguingly, tumors that are enriched for defects in DNA repair process were impressively susceptible to poly(ADP-ribose) polymerase (PARP) inhibitors, most prominent examples represent BRCA1- or BRCA2-deficient cells [48, 49]. Nevertheless, also BRCA-independent defects in HR-mediated DNA repair e.g. by downregulation of *protein phosphatase 2 regulatory subunit B alpha* (*PPP2R2A*) in non-small cell lung cancer cells promoted increased sensitivity to PARP inhibitors *in vitro* and *in vivo* [50]. Whether compromised DNA repair caused by *PPP4R2* inactivation may contribute to enhanced sensitivity to targeted therapy with e.g. PARP inhibitors remains to be elucidated. Further studies will determine the potential of *PPP4R2* deficiency as possible new target for AML therapy.

## MATERIALS AND METHODS

### Primary patient samples

Diagnostic BM and/or peripheral blood (PB) samples from AML patients (*n* = 85) and healthy BM controls (*n* = 8) were analyzed. Patients were enrolled into different consecutive AMLSG multicenter treatment trials [AML HD98A, NCT00146120 (*n* = 5); AML HD98B [51] (*n* = 4); AMLSG 06-04, NCT001512255 (*n* = 4); AMLSG 07-04, NCT00151242 (*n* = 57); AMLSG BiO, NCT01252485 (*n* = 4); AML 43, NCT00121303 (*n* = 2); RATIFY, NCT00651261 (*n* = 1); AMLSG-R1, NCT00744081 (*n* = 1); no study (*n* = 1); unknown (*n* = 6)]. All patients gave informed consent for treatment and genetic analysis according to the Declaration of Helsinki.

### Cell line models

BM from C57BL/6J wildtype mice was harvested and enriched for lineage-negative cells [Lin^-^, cells negative for lineage markers CD3, CD4, CD11b, CD19, TCRγ/δ, (BD Biosciences, Franklin Lakes, NJ, USA), CD8a, NK1.1, Gr-1, TCRα/β, Ter119 (eBioscience, San Diego, CA, USA)] using Dynabeads Sheep anti-rat IgG (Thermo Fisher Scientific, Waltham, MA, USA). Lin^-^ BM cells were cultured in StemSpan SFEM (StemCell Technologies, Vancouver, Canada) supplemented with 10 ng/ml thrombopoietin, 6 ng/ml interleukin (IL-)3, 10 ng/ml IL-6, 100 ng/ml stem cell factor, and 60 ng/ml granulocyte-colony stimulating factor (PeproTech, Rocky Hill, NJ, USA).

BM cells from C57BL/6J wildtype mice transformed by *MLLT3-KMT2A* transduction were cultured in IMDM+GlutaMAX (Thermo Fisher Scientific, Waltham, MA; USA) supplemented with 10% FBS (Hyclone, Thermo Fisher Scientific, Waltham, MA, USA), 1 mM sodium pyruvate (Thermo Fisher Scientific, Waltham, MA; USA), 0.002% 1thioglycerol (Sigma Aldrich, St. Louis, MO, USA), 1% IL-3 supernatant, and 1% penicillin/streptomycin (PAN-Biotech GmbH, Aidenbach, Germany). Hematopoietic subpopulations from C57BL/6J mice were sorted using fluorescent activated cell sorting as previously published [52].

Human leukemic cell line MEG-01 (DSMZ, Braunschweig, Germany) was cultured in RPMI1640 (biochrom, Berlin, Germany) supplemented with 10% FBS (Sigma Aldrich, St. Louis, MO, USA), 1% penicillin/streptomycin (PAN-Biotech GmbH, Aidenbach, Germany), and 2 mM L-glutamine (PAN-Biotech GmbH, Aidenbach, Germany).

### Phosphoprotein flow cytometry

Cells with either *Ppp4r2* knockdown/*PPP4R2* re-expression or control were exposed to 2 Gy IR or not followed by fixation and permeabilization at indicated time post IR with BD Cytofix/Cytoperm Buffer and BD Permeabilization Buffer Plus, respectively (Fixation/Permeabilization Solution Kit, BD Biosciences, Franklin Lakes, NJ, USA).

For measurement of pKAP1, cells were incubated with anti-pKAP1 antibody (S824; Bethyl Laboratories, Montgomery, TX, USA) and subsequently stained with anti-Dylight 649 donkey anti-rabbit IgG (BioLegend, San Diego, CA, USA).

Determination of γH2AX was performed by incubation with anti-phospho-Histone H2A.X antibody (Ser139; Clone JBW 301; Merck Millipore, Darmstadt, Germany) followed by staining with anti-Mouse IgG1 eFluor^®^ 660 (eBioscience, San Diego, CA, USA).

Cells were acquired by flow cytometry (FACSCalibur, BD Biosciences, Franklin Lakes, NJ, USA) and analyzed by Flow Jo Software (FlowJo, Ashland, OR, USA).

### Apoptosis analysis

Cells with either *Ppp4r2* knockdown/*PPP4R2* re-expression or control were exposed to IR with 2 Gy or not. At indicated time post IR, apoptotic cells were determined using the AnnexinV Apoptosis Detection Kit I (BD Biosciences, Franklin Lakes, NJ, USA) according to the manufacturer's instructions. Briefly, cells were washed twice with cold PBS and double-stained with APC AnnexinV (BD Biosciences, Franklin Lakes, NJ, USA) and 7-AAD. Data were acquired by flow cytometry (FACSCalibur, BD Biosciences, Franklin Lakes, NJ, USA) and analyzed with Flow Jo Software (FlowJo, Ashland, OR, USA).

### Colony formation assay (CFC-assay)

Murine BM cells (Lin^-^, *MLLT3-KMT2A*) with either *Ppp4r2* knockdown or control were plated in duplicates with 1 × 10^3^ cells per plate in methylcellulose MethoCult M3434 (StemCell Technologies, Vancouver, Canada) and colony forming cells were counted after 7 days.

Human leukemic MEG-01 cells with ectopic expression of *PPP4R2* or control were plated in duplicates with 300 cells per plate in methylcellulose MethoCult H4330 (StemCell Technologies, Vancouver, Canada) and colonies were counted after 10–14 days.

### Cell proliferation analysis (MTS-assay)

Cell viability and proliferation was assessed by determination of metabolic activity using the CellTiter 96^®^ AQ_ueous_ One Solution Cell Proliferation Assay (Promega, Madison, WI, USA). 2 × 10^4^ cells/ml with either *Ppp4r2* knockdown/*PPP4R2* re-expression or control were cultured in triplicates and examined daily according to the manufacturer's instructions.

### Analysis of gene expression profiling (GEP) data

A previously published GEP data set of our group comprising 436 AML cases accessible at gene expression omnibus (GEO accession GSE22778) was available for further studies [53]. Analyses were performed as previously reported by ClassComparison analysis using BRB Array Tools Version 3.8.0 – Beta_2 Release of BRB-Array Tools and using R, version 2.9.0 [54]. Pathway enrichment analysis was performed using the Molecular Signatures Database (MSigDB, http://broadinstitute.org/gsea/index.jsp).

### Whole exome sequencing (WES)

Whole exome sequencing was performed on five diagnosis and remission samples from cases with 3p CDR. DNA libraries were generated from 50 ng DNA using the Nextera Rapid Capture Expanded Exome kit (Illumina, San Diego, CA, USA) and the Nextera Rapid Capture Exome kit (Illumina, San Diego, CA, USA) according to the manufacturer's instructions. Pooled DNA libraries were sequenced on an Illumina HiSeq2000 with the 200-cycle TruSeq SBS v3 kit (Illumina, San Diego, CA, USA). Following de-multiplexing the paired-end sequences were aligned to the human reference genome hg19 with BWA-MEM23. BAM files were sorted and indexed using SortSam and BuildBamIndex (both Picard 1.138, http://picard.sourceforge.net). Variants were called using VarScan2 (http://varscan.sourceforge.net) by comparing tumor samples to the matching remission sample and annotated using ANNOVAR. Intronic variants, synonymous variants, variants with less than 2 tumor variant supporting reads in forward and reverse direction, variants with an entry in dbSNP13827 (http://www.ncbi.nlm.nih.gov/SNP), but not in COSMIC28 (http://cancer.sanger.ac.uk/cosmic), variants located in segmental duplication areas, variants which are located in polymer-repeat regions, and variants with a variant allele frequency <10% were filtered out. Pathway enrichment analysis was performed using the Molecular Signatures Database (MSigDB, http://broadinstitute.org/gsea/index.jsp). Statistical significance was assessed using a nonparametric Mann-Whitney test.

### Statistical analysis

Data are represented by each individual data point and the mean or mean ± SD, and number of replicates is indicated in the respective figure legend. Statistical analyses were carried out using unpaired two-tailed *t*-test or multiple *t*-tests corrected for multiple comparisons using the Holm-Sidak method (Prism 6, GraphPad Software, La Jolla, CA, USA). A *p*-value ≤ 0.05 was considered significant, *p ≤ 0.05, **p ≤ 0.01, ***p ≤ 0.001, ****p ≤ 0.0001.

## SUPPLEMENTARY MATERIALS FIGURES AND TABLES





## Abbreviations

*ABL1*: *ABL proto-oncogene 1*
AML: acute myeloid leukemia
AMLSG: AML Study Group
*B2M*: *beta-2 microglobulin*
BM: bone marrow
*BRCA*: *breast cancer*
*CDC27*: *cell division cycle 27*
CDR: commonly deleted region
CK: complex karyotype
CLP: common lymphoid progenitor(s)
CMP: common myeloid progenitor(s)
CN: cytogenetically normal
COSMIC: Catalogue of Somatic Mutations in Cancer
*CTBP2*: *C-terminal binding protein 2*
DDR: DNA damage response
*DNMT3A*: *DNA methyltransferase 3 alpha*
DSB: double strand break(s)
*EIF4E3*: *eukaryotic translation factor 4E family member 3*
*ERCC3*: *ERCC excision repair 3*
FISH: fluorescence *in situ* hybridization
*FLT3*: *fms related tyrosine kinase 3*
*FOXO3*: *forkhead box O3*
*FOXP1*: *forkhead box P1*
*GATA2*: *GATA binding protein 2*
GEP: gene expression profiling
GMP: granulocyte monocyte progenitor(s)
*GPR27*: *G protein-coupled receptor 27*
*GXYLT2*: *glucoside xylosyltransferase 2*
HR: homologous recombination
HSPC: hematopoietic stem and progenitor cell(s)
IL: interleukin
*KMT2A*: *lysine methyltransferase 2A*
Lin^-^: lineage-negative
LSK: Lin^-^Sca^+^cKit^+^
MEP: megakaryocytic erythroid progenitor(s)
*MLLT3*: *myeloid/lymphoid or mixed-lineage leukemia translocated to 3*
*MSH*: *mutS homolog*
*MSH2*: *mutS homolog 2*
*NFKB1*: *nuclear factor kappa B subunit 1*
NHEJ: non-homologous end-joining
*NPM1*: *nucleophosmin*
*NRAS*: *NRAS proto-oncogene GTPase*
*NUP98*: *nucleoporin 98*
PARP: poly(ADP-ribose) polymerase
PB: peripheral blood
pKAP1: phosphorylated KRAB-domain associated protein 1
pP53: phosphorylated P53
*PPP2R2A*: *protein phosphatase 2 regulatory subunit B alpha*
PPP4: protein phosphatase 4
*PPP4R2*: *protein phosphatase 4 regulatory subunit 2*
*PROK2*: *prokineticin 2*
*PTPN12*: *protein tyrosine phosphatase non-receptor type 12*
qRT-PCR: quantitative real-time PCR
RPA2: replication protein A2
*RYBP*: *RING1 and YY1 binding protein*
*SHQ1*: *H/ACA ribonucleoprotein assembly factor*
SNP: single-nucleotide polymorphism
*SUMO1*: *small ubiquitin-like modifier 1*
TCGA: The Cancer Genome Atlas
*TP53BP2*: *tumor protein 53 binding protein 2*
WES: whole exome sequencing
γH2AX: phosphorylated histone variant H2AX.
