# Supplementary material for: Protein phosphatase 4 regulatory subunit 2 (PPP4R2) is recurrently deleted in acute myeloid leukemia and required for efficient DNA double strand break repair
Source: Oncotarget. 2017 Sep 21;8(56):95038–53. doi: 10.18632/oncotarget.21119 (PMC5707003; doi:10.18632/oncotarget.21119)
Supplement: Supplementary file 1 [file oncotarget-08-95038-s001.pdf]

Protein phosphatase 4 regulatory subunit 2 (PPP4R2) is recurrently deleted in acute myeloid leukemia and required for efficient DNA double strand break repair

SUPPLEMENTARY MATERIALS

DNA/RNA isolation, cDNA synthesis, and quantitative real-time PCR (qRT-PCR)

RNA was extracted using RNeasy or miRNeasy Mini Kit (Qiagen, Hilden, Germany), or in parallel with DNA extraction using AllPrep Mini Kit (Qiagen, Hilden, Germany). RNA was reverse-transcribed using TaqMan Reverse Transcription Reagents (Thermo Fisher Scientific, Waltham, MA; USA). To quantify gene expression, cDNA was added to TaqMan Universal PCR Mastermix Kit (Thermo Fisher Scientific, Waltham, MA, USA) mixed with a primer/probe set [TaqMan Assays for human *PPP4R2* (Hs00752559_sH), murine *Ppp4r2* (Mm008086006_s1), *Ppp4r1* (Mm00455357_m1), *Ppp4r3a* (Mm01337081_m1), and *Ppp4r3b* (Mm00506177_m1; Thermo Fisher Scientific, Waltham, MA, USA); housekeeping genes human *beta-2 microglobulin B2M* (B2M-fw 5′- GAGTATGCCTGCCGTGTG-3′, B2M-rev 5′- AATCCAAATGCGGCATCT-3′, B2M-probe 5′-CCTCCATGATGCTGCTTACATGTCTC-3′), murine *beta-actin* (Thermo Fisher Scientific, Waltham, MA, USA)]. Gene expression analyses were performed at the 7900HT Fast Real-Time PCR System or QuantStudio 12K Flex Real-Time PCR System (Thermo Fisher Scientific, Waltham, MA, USA) with default thermal cycling conditions. Results were analyzed using the standard curve method or $2^{-\Delta\Delta Ct}$ method, respectively.

PPP4R2 mutation screening in AML patients by Sanger sequencing

Coding region of *PPP4R2* (NCBI Reference Sequence NM_174907.3) was amplified by polymerase-chain reaction (PCR) with exon-flanking primers (Supplementary Table 2). Purified PCR products were subjected to Sanger sequencing according to standard procedures.

Lentivirus production and infection for *Ppp4r2* knockdown or *PPP4R2* re-expression

We used lentivirus-based expression systems to knock down *Ppp4r2* or re-express *PPP4R2*. Knockdown experiments were conducted by expression of small hairpin RNAs (shRNAs) targeting *Ppp4r2* cloned into pLKO.1-puro vector (*Ppp4r2*-sh3, TRCN0000114143; *Ppp4r2*-sh4, TRCN0000114144; GE Healthcare Dharmacon, Lafayette, CO, USA) or a scrambled sequence as control (pLKO.1-scrambled-sh; Open Biosystems/GE Healthcare Dharmacon, Lafayette, CO, USA). For overexpression experiments, lentiviral expression vector pLOC containing the open reading frame (ORF) of *PPP4R2* (*PPP4R2*-ORF, PLOHS_ccsbBEn_05060; GE Healthcare Dharmacon, Lafayette, CO, USA), or *RFP* as control (*RFP*-control; GE Healthcare Dharmacon, Lafayette, CO, USA) were used. To produce lentivirus, 293T cells (DSMZ, Braunschweig, Germany) were co-transfected with psPAX2 (Addgene, Cambridge, MA, USA) and pMD2.G (Addgene, Cambridge, MA, USA) using TransIT-LT1 transfection reagent (Mirus Bio, Madison, WI, USA). Viral supernatant was harvested after 48 and 72 hours post transfection. To generate a cell line that stably expresses the *Ppp4r2* shRNA or *PPP4R2* ORF, cells were transduced with the respective lentivirus in the presence of 8 µg/ml polybrene (Merck Millipore, Darmstadt, Germany) and selected with puromycin (Sigma Aldrich, St. Louis, MO, USA) for knockdown experiments (0.5-1 µg/ml, murine Lin^- BM cells; 2 µg/ml, murine *MLLT3-KMT2A* Lin^- BM cells) or blasticidin (Thermo Fisher Scientific, Waltham, MA, USA) for overexpression experiments (5 µg/ml, MEG-01).

Western blot analysis

To obtain whole cell protein extracts, cells were lysed in lysis buffer (150 mM NaCl, 20 mM Tris, 5 mM EDTA, 1% TritonX, 10% glycerol) supplemented with Halt Phosphatase Inhibitor Cocktail (Thermo Fisher Scientific, Waltham, MA, USA) and Protease Arrest (G-Biosciences, St. Louis, MO, USA), or Laemmli buffer (50 mM Trizma base pH 6.8, 4% SDS, 20% glycerol, 0.02% bromphenolblue). For acid histone extraction, cells were lysed in cytolysis buffer (10 mM HEPES, 50 mM NaCl, 0.25 M sucrose, 0.1 mM EDTA, 0.5% Triton X) supplemented with Halt Phosphatase Inhibitor Cocktail (Thermo Fisher Scientific, Waltham, MA, USA) and Protease Arrest (G-Biosciences, St. Louis, MO, USA).

Nuclei were resuspended in histone extraction buffer (0.25 M HCl, 5% glycerol). Histones were precipitated with acetone and dissolved in water. 10-30 µg total protein or 10 µg histones were run on NuPAGE Novex Bis-Tris or Tris-Acetate gels (Thermo Fisher Scientific, Waltham, MA, USA). Separated proteins were transferred onto PVDF membrane (Merck Millipore, Darmstadt, Germany). Following blocking with 5% milk (Roth, Karlsruhe, Germany) or 2% chicken egg albumin (Sigma Aldrich, St. Louis, MO, USA), membranes were incubated with primary antibody against PPP4R2 (Bethyl Laboratories, Montgomery, TX, USA; abcam, Cambridge, UK; Thermo Fisher Scientific, Waltham, MA, USA), phospho-KAP1, phospho-RPA2 (Bethyl Laboratories, Montgomery, TX, USA), phospho-P53 (Ser15), GAPDH (Cell Signaling, Danvers, MA, USA), H2AX (abcam, Cambridge, UK), phospho-Histone H2A.X antibody (Ser139; Clone JBW 301; Merck Millipore, Darmstadt, Germany). After incubation with secondary antibody ECL Rabbit or Mouse IgG HRP-linked whole Ab (GE Healthcare, Lafayette, CO, USA), protein bands were detected using ECL solution and autoradiography films (GE Healthcare, Little Chalfont, UK) or Lumigen ECL Ultra (Southfield, MI, USA) and a chemiluminescence detection system (Vilber Lourmat, Eberhardzell, Germany). Antibodies were removed from membranes with Restore PLUS Western Blot Stripping Buffer (Thermo Fisher Scientific, Waltham, MA, USA) to reprobe and detect several proteins. Densitometric analysis of protein bands was carried out using ImageJ software (Wayne Rasband, National Institutes of Health, Bethesda; USA).

REFERENCES

1. Bagger FO, Sasivarevic D, Sohi SH, Laursen LG, Pundhir S, Sønderby CK, Winther O, Rapin N, Porse BT. BloodSpot: a database of gene expression profiles and transcriptional programs for healthy and malignant haematopoiesis. Nucleic Acids Res. 2016; 44:D917–24. https://doi.org/10.1093/nar/gkv1101.

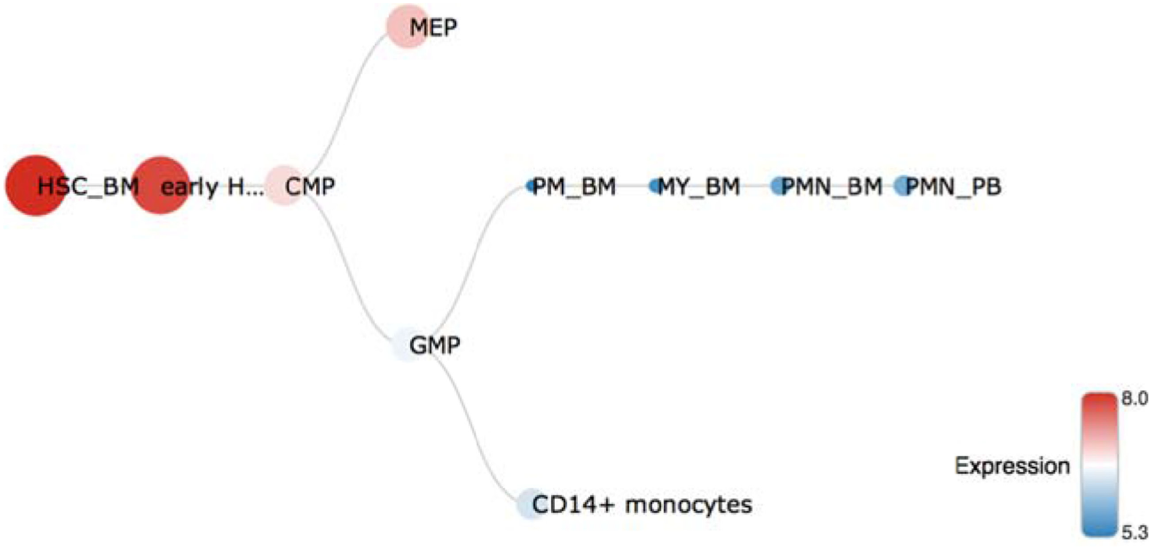

Supplementary Figure 1: *PPP4R2* is differentially expressed in human myelopoiesis. Hierarchical tree of *PPP4R2* expression in human hematopoietic cells based on microarray data (probeset 226317_at) [1]. HSC_BM, hematopoietic stem cells from bone marrow; early HPC_BM, hematopoietic progenitor cells from bone marrow; CMP, common myeloid progenitor cell; GMP, granulocyte monocyte progenitors; MEP, megakaryocyte-erythroid progenitor cell; PM_BM, promyelocyte from bone marrow; MY_BM, myelocyte from bone marrow; PMN_BM, polymorphonuclear cells from bone marrow; PMN_PB, polymorphonuclear cells from peripheral blood.

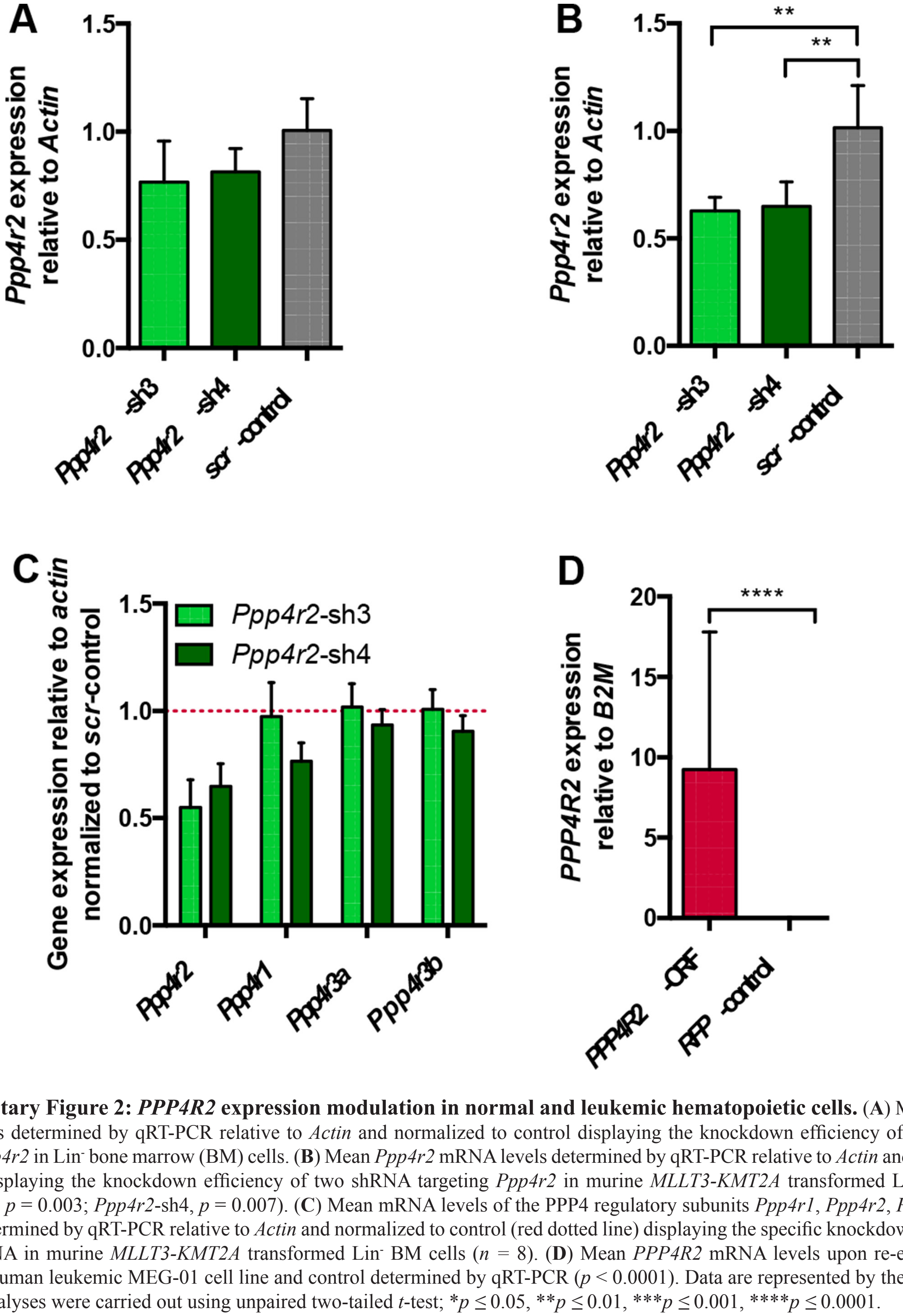

Supplementary Figure 2: *PPP4R2* expression modulation in normal and leukemic hematopoietic cells. (**A**) Mean *Ppp4r2* mRNA levels determined by qRT-PCR relative to *Actin* and normalized to control displaying the knockdown efficiency of two shRNA targeting *Ppp4r2* in Lin$^-$ bone marrow (BM) cells. (**B**) Mean *Ppp4r2* mRNA levels determined by qRT-PCR relative to *Actin* and normalized to control displaying the knockdown efficiency of two shRNA targeting *Ppp4r2* in murine *MLLT3-KMT2A* transformed Lin$^-$ BM cells (*Ppp4r2*-sh3, $p = 0.003$; *Ppp4r2*-sh4, $p = 0.007$). (**C**) Mean mRNA levels of the PPP4 regulatory subunits *Ppp4r1*, *Ppp4r2*, *Ppp4r3a*, and *Ppp4r3b* determined by qRT-PCR relative to *Actin* and normalized to control (red dotted line) displaying the specific knockdown of *Ppp4r2* by two shRNA in murine *MLLT3-KMT2A* transformed Lin$^-$ BM cells ($n = 8$). (**D**) Mean *PPP4R2* mRNA levels upon re-expression of *PPP4R2* in human leukemic MEG-01 cell line and control determined by qRT-PCR ($p < 0.0001$). Data are represented by the mean ± SD. Statistical analyses were carried out using unpaired two-tailed *t*-test; $*p \leq 0.05$, $**p \leq 0.01$, $***p \leq 0.001$, $****p \leq 0.0001$.

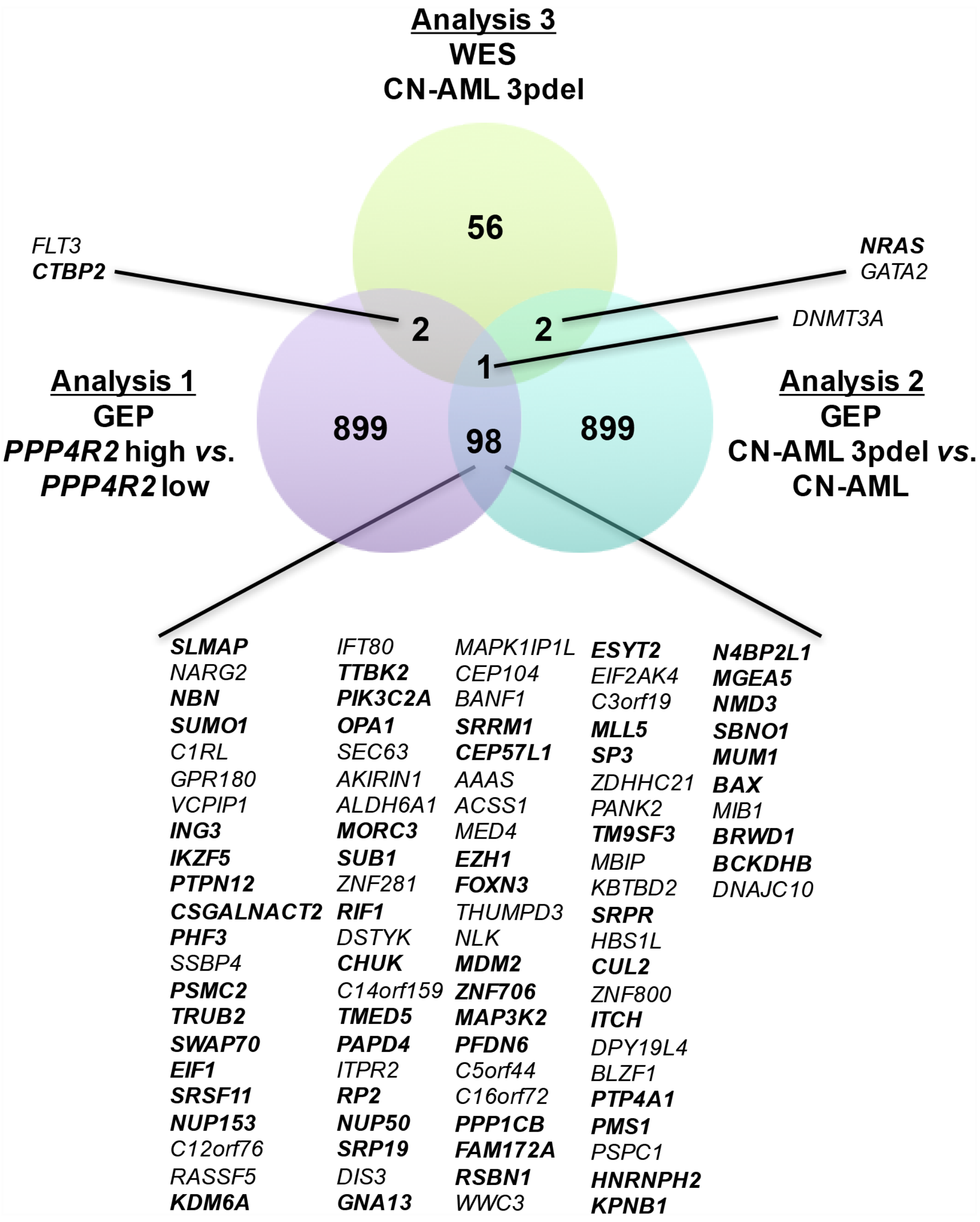

Supplementary Figure 3: Gene expression profiling (GEP) and whole exome sequencing (WES) of primary AML samples identified enriched gene sets associated with DNA repair. Venn diagram illustrating the overlap of the top 1000 most significant co-expressed genes of AML patients with high (*n* = 30) *versus* low (*n* = 30) global *PPP4R2* expression obtained by GEP (analysis 1), the top 1000 most significant co-expressed genes of CN-AML with 3p microdeletion (*n* = 9) *versus* without 3p microdeletion (*n* = 161) obtained by GEP (analysis 2), and the mutated genes of AML patients with 3p microdeletion (*n* = 5) detected by WES (analysis 3). Bold genes were identified in gene sets that are associated with processes in response to DNA damage.

Supplementary Table 1: Characteristics of CN-AML patients with 3p microdeletion (*n* = 10) and CK-AML patients with 3p microdeletion (*n* = 29). See Supplementary_Table 1

Supplementary Table 2: PCR primers used for *PPP4R2* amplification and mutation screening

Amplicon	Forward primer sequence 5′–3′	Reverse primer sequence 5′–3′
Exon 1	GAGGTGCTCGCTCTGTCG	AACCATCCGCCCTGAAAG
Exon 2	GGTGGTAATTTGGTTGAGAGGC	ATTCTCTAAGCGTGCCAAAGC
Exon 3	AATGAGGCATGATGTGTGCTC	TGGATAATCAATCATCAACCAGAC
Exon 4	AAATGACTCATCCATTTATTTCCC	CCCGAAACAGATCTTACTCAATG
Exon 5	TCAATGGGAATTTAACCCACC	GCAATGAGTGCCACCACAG
Exon 6	CATTCCACCTTGGGTGACAG	TTTCAGGCCCATGCCTTAC
Exon 7	TTAATTTGGTAAGGCATGGGC	GAAGCCAGTGCTCCTATAAACC
Exon 8	TCCTACTGGGAATTATTCATCAAA	CAGCATTATAAGGAGTTCCATTACG
Exon 9	TGTACCCGGCAGCACTGTA	AACTACTACTTACAGGGCCTTCGTT

Supplementary Table 3: Pathway enrichment analysis of the top 1000 most significant co-expressed genes of AML patients with high (n = 30) *versus* low (n = 30) global *PPP4R2* expression

Gene Set Name	# Genes in Gene Set (K)	Description	# Genes in Overlap (k)	k/K	*p*-value	FDR *q*-value
**PILON_KLF1_ TARGETS_DN**	1972	Genes down-regulated in erythroid progenitor cells from fetal livers of E13.5 embryos with KLF1 [GeneID = 10661] knockout compared to those from the wild type embryos.	239	0.1212	2.5E-109	1.18E-105
**DACOSTA_UV_ RESPONSE_VIA_ ERCC3_DN**	855	Genes down-regulated in fibroblasts expressing mutant forms of ERCC3 [GeneID = 2071] after UV irradiation.	143	0.1673	1.32E-82	3.11E-79
**DIAZ_CHRONIC_ MEYLOGENOUS_ LEUKEMIA_UP**	1382	Genes up-regulated in CD34+ [GeneID = 947] cells isolated from bone marrow of CML (chronic myelogenous leukemia) patients, compared to those from normal donors.	168	0.1216	1.84E-75	2.89E-72
**BUYTAERT_ PHOTODYNAMIC_ THERAPY_ STRESS_UP**	811	Genes up-regulated in T24 (bladder cancer) cells in response to the photodynamic therapy (PDT) stress.	131	0.1615	1.32E-73	1.56E-70
**GRAESSMANN_ APOPTOSIS_BY_ DOXORUBICIN_DN**	1781	Genes down-regulated in ME-A cells (breast cancer) undergoing apoptosis in response to doxorubicin [PubChem = 31703].	174	0.0977	1.26E-63	1.2E-60
**PUJANA_BRCA1_ PCC_NETWORK**	1652	Genes constituting the BRCA1-PCC network of transcripts whose expression positively correlated (Pearson correlation coefficient, PCC > = 0.4) with that of BRCA1 [GeneID = 672] across a compendium of normal tissues.	152	0.0920	6.24E-52	4.91E-49
**RODRIGUES_ THYROID_ CARCINOMA_ ANAPLASTIC_UP**	722	Genes up-regulated in anaplastic thyroid carcinoma (ATC) compared to normal thyroid tissue.	100	0.1385	1.03E-49	6.96E-47
**MILI_ PSEUDOPODIA_ HAPTOTAXIS_UP**	518	Transcripts enriched in pseudopodia of NIH/3T3 cells (fibroblast) in response to haptotactic migratory stimulus by fibronectin, FN1 [GeneID = 2335].	86	0.1660	3.09E-49	1.83E-46
**JOHNSTONE_ PARVB_ TARGETS_3_DN**	918	Genes down-regulated upon overexpression of PARVB [GeneID = 29780] in MDA-MB-231 cells (breast cancer) cultured in 3D Matrigel only.	108	0.1176	1.08E-46	5.65E-44
**ACEVEDO_LIVER_ CANCER_UP**	973	Genes up-regulated in hepatocellular carcinoma (HCC) compared to normal liver samples.	109	0.1120	4.57E-45	2.16E-42

Supplementary Table 4: Pathway enrichment analysis of the top 1000 most significant co-expressed genes of CN-AML with 3p microdeletion (*n* = 9) *versus* without 3p microdeletion (*n* = 161)

Gene Set Name	# Genes in Gene Set (K)	Description	# Genes in Overlap (k)	k/K	*p*-value	FDR *q* -value
PILON_KLF1_TARGETS_DN	1972	Genes down-regulated in erythroid progenitor cells from fetal livers of E13.5 embryos with KLF1 [GeneID = 10661] knockout compared to those from the wild type embryos.	222	0.1126	1.67E-94	7.88E-91
DIAZ_CHRONIC_ MEYLOGENOUS_ LEUKEMIA_UP	1382	Genes up-regulated in CD34+ [GeneID = 947] cells isolated from bone marrow of CML (chronic myelogenous leukemia) patients, compared to those from normal donors.	175	0.1266	1.37E-81	3.24E-78
JOHNSTONE_PARVB_ TARGETS_3_DN	918	Genes down-regulated upon overexpression of PARVB [GeneID = 29780] in MDA-MB-231 cells (breast cancer) cultured in 3D Matrigel only.	140	0.1525	2.14E-75	3.38E-72
GRAESSMANN_APOPTOSIS_ BY_DOXORUBICIN_DN	1781	Genes down-regulated in ME-A cells (breast cancer) undergoing apoptosis in response to doxorubicin [PubChem = 31703].	183	0.1028	1.46E-70	1.73E-67
PUJANA_BRCA1_PCC_ NETWORK	1652	Genes constituting the BRCA1-PCC network of transcripts whose expression positively correlated (Pearson correlation coefficient, PCC >= 0.4) with that of BRCA1 [GeneID = 672] across a compendium of normal tissues.	172	0.1041	5.52E-67	5.21E-64
KINSEY_TARGETS_OF_ EWSR1_FLII_FUSION_UP	1278	Genes up-regulated in TC71 and EWS502 cells (Ewing's sarcoma) by EWSR1-FLI1 [GeneID = 2130;2314] as inferred from RNAi knockdown of this fusion protein.	142	0.1111	1.83E-58	1.44E-55
PUJANA_ATM_PCC_ NETWORK	1442	Genes constituting the ATM-PCC network of transcripts whose expression positively correlated (Pearson correlation coefficient, PCC >= 0.4) with that of ATM [GeneID=472] across a compendium of normal tissues.	148	0.1026	1.59E-56	1.08E-53
DACOSTA_UV_RESPONSE_ VIA_ERCC3_DN	855	Genes down-regulated in fibroblasts expressing mutant forms of ERCC3 [GeneID = 2071] after UV irradiation.	107	0.1251	7.48E-49	4.42E-46
NUYTTEN_NIPP1_TARGETS_ DN	848	Genes down-regulated in PC3 cells (prostate cancer) after knockdown of NIPP1 [GeneID = 5511] by RNAi.	100	0.1179	2.48E-43	1.3E-40
MILI_PSEUDOPODIA_ HAPTOTAXIS_UP	518	Transcripts enriched in pseudopodia of NIH/3T3 cells (fibroblast) in response to haptotactic migratory stimulus by fibronectin, FN1 [GeneID = 2335].	79	0.1525	1.79E-42	8.45E-40

Supplementary Table 5: Pathway enrichment analysis of the mutations detected by whole exome sequencing of AML patients with 3p microdeletion (*n* = 5)

Gene Set Name	# Genes in Gene Set (K)	Description	# Genes in Overlap (k)	k/K	*p*-value	FDR *q*-value
CAIRO_ HEPATOBLASTOMA_ CLASSES_UP	605	Genes up-regulated in robust Cluster 2 (rC2) of hepatoblastoma samples compared to those in the robust Cluster 1 (rC1).	8	0.0132	1.37E-6	6.5E-3
GRESHOCK_CANCER_ COPY_NUMBER_UP	323	Genes from common genomic gains observed in a meta analysis of copy number alterations across a panel of different cancer cell lines and tumor samples.	6	0.0186	4.62E-6	7.79E-3
KEGG_PATHWAYS_IN_ CANCER	328	Pathways in cancer	6	0.0183	5.04E-6	7.79E-3
REACTOME_SIGNALING_ BY_ERBB4	90	Genes involved in Signaling by ERBB4	4	0.0444	6.59E-6	7.79E-3
KARLSSON_TGFB1_ TARGETS_DN	207	Genes down-regulated by TGFB1 [GeneID=7040] in MEF cells (embryonic fibroblast) via TGFB1R [GeneID = 7046].	5	0.0242	8.56E-6	8.1E-3
BAE_BRCA1_TARGETS_DN	32	Genes concordantly down-regulated in DU-145 and MCF-7 cells (lprostate, breast cancer) upon expression of BRCA1.	3	0.0938	1.07E-5	8.46E-3
DACOSTA_UV_RESPONSE_ VIA_ERCC3_DN	855	Genes down-regulated in fibroblasts expressing mutant forms of ERCC3 [GeneID = 2071] after UV irradiation.	8	0.0094	1.71E-5	1.16E-2
IVANOVA_HEMATOPOIESIS_ STEM_CELL_AND_ PROGENITOR	681	Genes in the expression cluster 'HSC and Progenitors Shared': up-regulated in hematopoietic stem cells (HSC) and progenitors from adult bone marrow and fetal liver.	7	0.0103	3.32E-5	1.96E-2
KEGG_ENDOMETRIAL_ CANCER	52	Endometrial cancer	3	0.0577	4.69E-5	2.47E-2
MARTORIATI_MDM4_ TARGETS_FETAL_LIVER_ DN	514	Genes down-regulated in non-apoptotic tissues (fetal liver) after MDM4 [GeneID = 4194] knockout.	6	0.0117	6.27E-5	2.84E-2
