# Supplementary material for: Protein phosphatase 4 regulatory subunit 2 (PPP4R2) is recurrently deleted in acute myeloid leukemia and required for efficient DNA double strand break repair
Source: Oncotarget. 2017 Sep 21;8(56):95038–53. doi: 10.18632/oncotarget.21119 (PMC5707003; doi:10.18632/oncotarget.21119)
Supplement: Supplementary file 2 [file oncotarget-08-95038-s002.docx]

**Supplementary Table 1:** **Characteristics of CN-AML patients with 3p microdeletion (n=10) and CK-AML patients with 3p microdeletion (n=29)**

| Patient | Karyotype | Molecular genetics/cytogenetics |
| --- | --- | --- |
| 1 | CN-AML | *NPM1*^mut^, *FLT3*-ITD^neg^ |
| 2 | CN-AML | *NPM1*^mut^, *FLT3*-ITD^pos^ |
| 3 | CN-AML | *NPM1*^mut^, *FLT3*-ITD^neg^ |
| 4 | CN-AML | *NPM1*^mut^, *FLT3*-ITD^pos^ |
| 5 | CN-AML | *NPM1*^mut^, *FLT3*-ITD^pos^ |
| 6 | CN-AML | *NPM1*^mut^, *FLT3*-ITD^pos^ |
| 7 | CN-AML | *NPM1*^mut^, *FLT3*-ITD^neg^ |
| 8 | CN-AML | *NPM1*^wt^, *FLT3*-ITD^neg^ |
| 9 | CN-AML | *NPM1*^wt^, *FLT3*-ITD^neg^ |
| 10 | CN-AML | *NPM1*^mut^, *FLT3*-ITD^pos^ |
| 11 | CK-AML | ~46,XY,add(3)(p13),add(5)(q11),add(6)(q2?7),-7,add(9)(p13),-16,add(17)(p11), iso(21)(p10),+mar[21]/46,XY[1] |
| 12 | CK-AML | 44,XY,add(3)(p1?2),-5,-6,-12,-13,-13,+3mar[2]/44-45,XY,add(3)(p1?2),-5,-6,-12,-14,+2-3mar[2] |
| 13 | CK-AML | 44,XYins(3)(q21;p14p23),-5,add(7)(q11),add(15)(p10),-16,add(17)(p11),-20,-22,+2mar[19] |
| 14 | CK-AML | 45,XX,-3,del(5)(q13q33),der(6)t(3;6)(q11;q27),-7,+8,del(12)(p11p13)[11] |
| 15 | CK-AML | 45,XY,der(3)inv(3)(q21q26.2)del(3)(p11),-7,add(8p),-11,+1mar[17] |
| 16 | CK-AML | 44-45,XY,-3,del(5q),der(6)t(3;6)(q21;p23),-14,-15,-16,-21,-22,-22,+5-6mar[22] |
| 17 | CK-AML | 43,XY,add(1)(p36),del(1)(q32),del(2)(q22q37),-3,-5,del(6)(q12q27),-7,del(12)(q22q37),-13,-16,-17,del(20)(q11q13),+1-3mar[18] |
| 18 | CK-AML | 44-46,XX,-3,del(5)(q11),del(6)(q?),add(7)(q22),del(9)(q13),-11,idic(11)(p11),add(12)(p12),-16,-17,+2-4mar |
| 19 | CK-AML | 45~47,XX,del(3)(p12p23),der(5;17)(p10,q10),-7,+8,+11,-18,-22,+2~3mar[20] |
| 20 | CK-AML | 45,XX,del(3)(p13p23),-9,del(11)(q22),del(12)(p11p13),-17,+mar[10] |
| 21 | CK-AML | 45,XY,del(3)(p11),add(5)(q13),der(13)t(1;13)(p31;p11),17,add(21)(q22)[20] |
| 22 | CK-AML | 46-48,XX,del(3)(p12p24),del(5)(q?),del(10)(q24),-13,-17,+2-4mar[13] |
| 23 | CK-AML | 46,XX,del(1)(p36),add(3)(q13),del(3)(p13p21),-5,add(6)(p21),del(7)(q11),-9,add(10)(p15),add(14)(q32),del(17)(p13),+2mar,+r[10] |
| 24 | CK-AML | ~59,X,-X,-X,-1,der(3)add(q2?5)add(p13),-4,-5,-6,-7,-12,-14,-16,-17,2xadd(21)(p11),+1~2mar |
| 25 | CK-AML | 45,XX,-3,?del(3)(q21),del(5)(q15q33),-7,-12,-13,-17,+mar1-4[15] |
| 26 | CK-AML | 47,XY,add(3)(p21),del(5)(q?),+6,-7,+8,add(12)(p11),-15,+mar |
| 27 | CK-AML | 42,XY,add(1)(q32),-2,-3,del(5)(q13q33),-7,-17,-18,-21,+2mar/42,XY,idem,-22,+3mar |
| 28 | CK-AML | 45,XY,del(3)(p13p23),-5,+8,-12,-15,add(17)(q23),add(21)(p11),+mar[20] |
| 29 | CK-AML | 49,XY,der(3)add(3)(p21)del(3)(q21q26),add(5)(q15),+8,+10, del(11)(p11),-12,+13,del(17)(p11),+21[5] |
| 30 | CK-AML | 46,Y,-X,t(3;8)(q13;p21),t(4;6)(q12;q25),-7,add(18)(q21),+mar1,+mar2[10] |
| 31 | CK-AML | 44,XY,del(3)(p12p14),del(5)(q31),-7,del(8)(p12),-11,-16,+mar[9]/46,XY[1] |
| 32 | CK-AML | 37~43,X,-X,del(3)(p13p2?4),-5,inc[cp11] |
| 33 | CK-AML | 46,XX,der(1),der(1),der(2),der(3)add(3)(q11)add(3)(p?),add(5)(q15),-7,+i(9)(p10),del(12)(p11p13),-16,-17,del(18)(q21),+2mar [16] |
| 34 | CK-AML | 44,XY,-3,-5,-7,inv(9)(p11q13)c,-12,-14,-15,-17,+mar1,+mar2,+mar3,+mar4,+mar5,inc[17] |
| 35 | CK-AML | 72,XXXX, including -3,-7,-9,-10,-11,-13,-15,-17,-19,-20,add(21p),-22,+mar |
| 36 | CK-AML | 44,XX,del(2)(q31q35),add(3)(p11),-4,del(5)(q13q33),-11,dup(11)(q21q23),-14,del(17)(q21),del(18)(q21),+mar[15] |
| 37 | CK-AML | 62,XYY,del(3)(p11),-4,-5,del(5)(q15q33),-7,-9,-10,+11,-12,-15,-17,-19,-20,-22,+1-4mar,+dmin[19] |
| 38 | CK-AML | 46,XY,-2,add(2)(q37),del(3)(p11p21),add(6)(p21),+8,-9,del(22)(q11),+mar[12] |
| 39 | CK-AML | 44~46,XX,+del(1)(q21),der(3)add(3)(p13)add(3)(q21),-5,-7,+8,-17,+2~3mar,inc[cp12]/46,XX[1] |
| CN, cytogenetically normal; CK, complex karyotype; AML, acute myeloid leukemia; *NPM1*, *nucleophosmin*; *FLT3*; *fms related tyrosine kinase 3*; mut, mutated; wt, wildtype; ITD, internal tandem duplication; neg, negative; pos, positive. | | |
